# Inner Mongolian Cashmere Goat Secondary Follicle Development Regulation Research Based on mRNA-miRNA Co-analysis

**DOI:** 10.1038/s41598-020-60351-5

**Published:** 2020-03-11

**Authors:** Wenjing Han, Feng Yang, Zhihong Wu, Fuqiang Guo, Junjie Zhang, Erhan Hai, Fangzheng Shang, Rui Su, Ruijun Wang, Zhiying Wang, Zhihong Liu, Yanhong Zhao, Zhixin Wang, Yanjun Zhang, Jinquan Li

**Affiliations:** 1 College of Animal Science, Inner Mongolia Agricultural University, Hohhot, Inner Mongolia Autonomous Region, 010018 Hohhot, China; 2 Chemistry and Life Science College,Chi Feng University, Chi Feng, Inner Mongolia Autonomous Region, 024000 Hohhot, China; 3 Key Laboratory of Animal Genetics, Breeding and Reproduction in Inner Mongolia Autonomous Region, Hohhot, Inner Mongolia Autonomous Region, 010018 Hohhot, China; 4 Engineering Research Center for Goat Genetics and Breeding, Inner Mongolia Autonomous Region, Hohhot, Inner Mongolia Autonomous Region, 010018 Hohhot, China; 5 Key Laboratory of Mutton Sheep Genetics and Breeding, Ministry of Agriculture, Hohhot, Inner Mongolia Autonomous Region, 010018 Hohhot, China

**Keywords:** Reporter genes, Data processing

## Abstract

Inner Mongolia cashmere goats, as an important part of animal husbandry production, play an important role in animal fiber industry. In recent years, scientific research has made a lot of explorations on the molecular regulation mechanism of hair follicle cycle growth, but few studies have been reported on the development of cashmere hair in fetal period. This study was based on the completion of 21 skin samples of mRNA and miRNA sequencing in 7 fetal periods (45 days, 55 days,65 days,75 days,95 days,115 days and 135 days) of the Inner Mongolia Cashmere goat. The target genes of miRNA associated with the development of secondary hair follicles in the cashmere goats were selected through the combination analysis of mRNA and miRNA data. Then the overexpression vector was constructed and the interaction between the miRNA and the target gene was identified by Dual-Luciferase Reporter Gene System. The function and interaction relationship of *chi*-miR-199a-5p and *TGF-β2* were verified by RT-qPCR and western blot at the level of the fibroblasts in Inner Mongolia Cashmere goat. It provides a theoretical basis for further study of miRNA and its target genes regulating the occurrence and development of skin hair follicles. As the result shows, the expression trends of 7 genes (*BAMBI, SMAD1, LTBP1, PPP2R1B, ID4, BMP8B* and *PITX2*) and 7 miRNA (*chi*-miR-17-5p, *chi*-miR-125b-3p, *chi*-miR-21-5p, *chi*-miR-143-5p and *chi*-miR-106b-5p) in the skin samples for the seven stages of the fetus were shown to be consistent with the sequencing results. the results of sequencing are reliable. The correlation coefficient of *TGF-β2* and *chi*-miR-199a-5p in fetal 45d-135d expression is −0.84, showing a strong negative correlation, The target relationship was preliminarily judged. The results of double luciferase vector report showed that *chi*-miR-199a-5p significantly decreased the expression of luciferase in *TGF-β2* 3′UTR, It is determined that there is a reciprocal relationship between them at a specific time. We transfected *chi*-miR199a-5p-FAM mimics into fibroblasts cultured *in vitro* from Inner Mongolia cashmere goats. After transfection, the cells were harvested to extract total RNA and protein. The mRNA and protein expression levels of *TGF-β2* in fibroblasts were detected by RT-qPCR and western blot. It was verified that *chi*-miR-199a-5p inhibited *TGF-β2* expression at both mRNA and protein translation levels in fibroblasts. At the same time, it was again proved that the *TGF-β2* gene is a target gene of chi-miR199a-5p.

## Introduction

The Inner Mongolia cashmere goats are an indispensable part of Inner Mongolia animal husbandry. The Inner Mongolia cashmere goats is a type of local cashmere goat mainly produced in the western region of Inner Mongolia where it is widely recognized as an animal of profound economic value. The cashmere produced by the Inner Mongolia cashmere goat is fine and slender, has a white texture and good luster, and is a high-grade textile material^1,2^.

The yield and quality of cashmere is controlled by the hair follicle, a complex skin accessory organ of the goat. The morphology and structure of the hair follicle controls hair growth with periodic growth characteristics (anagen, catagen, telogen)^3^. The cashmere goats coat has a different pelage type. The growth of wool is called primary follicles. The growth of the cashmere is called secondary follicles. There is a difference in the shape of different parts of the hair follicles, but all of them have the same basic structure^4,5^. The occurrence of hair follicles in the embryonic period involves a complex series of epidermis and dermis interactions, first by the initial signal from mesenchymal cells, inducing epidermal form hair germ. Then, the hair germ releases some factors inducing formation of the dermal fibroblast and the dermal papilla, where the dermal papilla releases a second signal of stimulation for proliferation and differentiation of the epithelial cell to form a complete structure of the hair follicle^4,6^. Therefore, epidermal cells and dermal fibroblasts must play an important role in hair follicle development. Differentiation and development of the hair follicle involves a series of signaling molecules. It is currently believed that most of these signaling molecules are part of the WNT signaling pathway, the TNF family, the TGF family, the FGF family, the MAPK signaling pathway, the SHH signaling pathway, the Notch signaling pathway, and other pathways^7^. In recent years, more studies have shown that miRNA also plays an important role in the occurrence and development of skin hair follicles^8,9^. miRNA is an important factor in regulating gene expression as it participates in cell proliferation, differentiation, metabolism, disease, and other important biological processes^10,11^. In 2006, two studies on the role of miRNA in the occurrence and development of murine skin hair follicles initiated the study on the effect of miRNA on hair follicle development^12,13^. Studies have predicted that target genes of let-7b and mir-24 are related to the growth of, the development of, and hair quality from the hair follicle^14^. Overexpression of miR-125b affects the expression level of mRNA related to follicular periodic changes^15^. Preliminarily data with the stable expression miR-206 after its transfection in secondary hair papilla cells showed that overexpression miR-206 had a certain inhibitory effect on the development of hair follicles in the growing period^16^.

The group has sequenced 21 samples of skin mRNA and miRNA collected during seven stages of the fetal period (45 days, 55 days, 65 days, 75 days, 95 days, 115 days, and 135 days) of the Inner Mongolia cashmere goat. In this study, the target genes of miRNA associated with the development of secondary hair follicles in the cashmere goats were selected through the combination of mRNA and miRNA data. The target was identified using the Dual-Luciferase Reporter Gene System. The functions of chi-miR-199a-5p were studied at the level of the fibroblasts in Inner Mongolia cashmere goats.

## Results

### MRNA and sRNA sequencing and quality assessment results

In order to ensure data quality, we control the original data before data processing, and reduce data impurities through data filtering. We filtered and screened raw data, The results showed that the coverage of clean reads Q20 of RNA was more than 90% (Table 1), and that of clean reads of microRNA was more than 98% (Table 2). The sequencing depth and coverage of samples meet the needs of this experiment and can be used for subsequent experiments.

**Table 1:** Obtain the RNA-Seq clean data.

Sample	Raw data	Q20(%)	Clean data	Q20(%)
FP-45d-1	10474685176	9415987952 (89.89%)	9205473650	8504592068 (92.39%)
FP-45d-2	11576565094	10401228718 (89.85%)	10442107411	9615872599 (92.09%)
FP-45d-3	11516657958	10417424323 (90.46%)	9297102448	8633474874 (92.86%)
FP-55d-1	11574536258	10458779016 (90.36%)	9984099030	9265615364 (92.80%)
FP-55d-2	10099252970	8833019818 (87.46%)	7981216720	7232763577 (90.62%)
FP-55d-3	14361793616	12887754687 (89.74%)	12675556365	11691886261 (92.24%)
FP-65d-1	10425039698	9366852432 (89.85%)	9148801979	8449997648 (92.36%)
FP-65d-2	11213896918	10093011637 (90.00%)	9502256844	8801158209 (92.62%)
FP-65d-3	11291800536	10268565021 (90.94%)	10097029771	9399156690 (93.09%)
FP-75d-1	3802435500	3561521649 (93.66%)	3723142144	3507969123 (94.22%)
FP-75d-2	4234597500	3973241698 (93.83%)	4145546791	3911512268 (94.35%)
FP-75d-3	3387849000	3173001276 (93.66%)	3315942774	3123835278 (94.21%)
FP-95d-1	3796614900	3559327755 (93.75%)	3716462474	3504404467 (94.29%)
FP-95d-2	3778704000	3544061516 (93.79%)	3696702322	3487323617 (94.34%)
FP-95d-3	3376559100	3147221091 (93.21%)	3296243262	3093396327 (93.85%)
FP-115d-1	3565607700	3335669909 (93.55%)	3486027899	3281285963 (94.13%)
FP-115d-2	3556965600	3319319387 (93.32%)	3477546153	3265864880 (93.91%)
FP-115d-3	3451999200	3245621202 (94.02%)	3387012229	3200797881 (94.50%)
FP-135d-1	3599433900	3370862722 (93.65%)	3524056472	3319484507 (94.19%)
FP-135d-2	3405073200	3196649921 (93.88%)	3337974518	3151203478 (94.40%)
FP-135d-3	3083014200	2886040524 (93.61%)	3019391433	2843133915 (94.16%)

**Table 2:** Obtain the miRNA-seq clean reads.

Sample	Raw reads	Clean reads	%
FP-45d	5253789	5253789	100%
FP-55d	7998727	7998727	100%
FP-65d	5172144	5172144	100%
FP-75d	9822658	9657611	98.3197%
FP-95d	11523639	11373163	98.6942%
FP-115d	9879903	9748726	98.6723%
FP-135d	12922560	12748752	98.6550%

### Analysis differences of mRNA and miRNA

**Figure 1:**
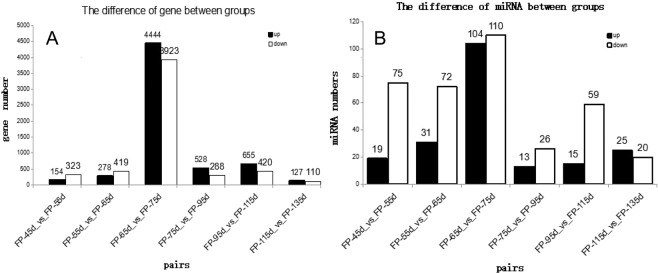
(**A**) The difference of gene between groups. (**B**) The difference of miRNA between groups. Pairs indicates pairwise comparisons; up indicates a significantly up-regulated gene; down indicates a significant down-regulation gene.

**Figure 2:**
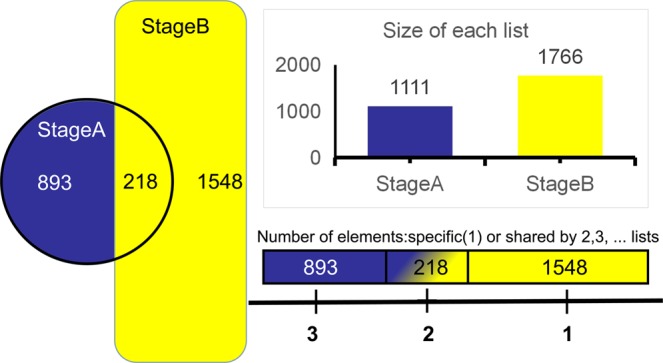
veen diagiam. The column diagram shows the total number of redundant elements in each input sample. The distribution map shows the distribution of all elements in each sample combination.

Two comparisons were made on the sequence data of mRNA and miRNA of Inner Mongolia cashmere goats at seven fetal stages (45d, 55d, 65d, 75d, 95d, 115d and 135d). Six sets of data were obtained: FP-45d_vs_FP-55d, FP-55d_vs_FP-65d, FP-65d_vs_FP-75d, FP-75d_vs_FP-95d, FP-95d_vs_FP-115d and FP-115d_vs_FP-135d (Figs. 1 and 2). Gene expression results (Fig. 1A) showed that there were differences in the number of gene expression among the groups, Among them, FP-65d_vs_FP-75d has the largest number of differentially expressed genes and the highest number of differentially expressed genes. There are 4444 up-regulated genes and 3923 down-regulated genes in the number of differentially expressed genes. The number of differentially expressed genes is as high as 8967, It is speculated that the occurrence and development of cashmere follicle will start in the first 65 to 75 days. The results of microRNAs expression (Fig. 1B) showed that there were differences among the groups, but the overall down-regulation of microRNAs expression was higher than the up-regulation of microRNAs, And in FP-65d_vs_FP-75d, the number of miRNA expression was the largest, the number of expression differences was the highest as well, The number of expression was upregulated 104 miRNA and down regulated miRNA 110, and the number of differentially expressed genes was as high as 214. It is speculated that microRNAs are involved in the initiation of hair follicle formation and development in cashmere goats between 65 and 75 days of gestation, and are mainly negatively regulated.

### Candidate gene screening

In order to screen candidate genes, the first three time points (45d, 55d and 65d) of differential genes were combined (stage A) and the later four time points samples (75d, 95d, 115d and 135d) differential genes were collected (stage B). Then we did Wien diagram for stage A and stage B (Fig. 2). It was found that the number of stage A differentially expressed genes was 1111 and stage B was 1766. The number of differentially expressed genes in the two groups was 218. Sifting out the differential genes in the two groups, the remaining 1548 differentially expressed genes are candidate genes associated with secondary hair follicle development.

### GO function analysis and KEGG Pathway analysis

**Figure 3:**
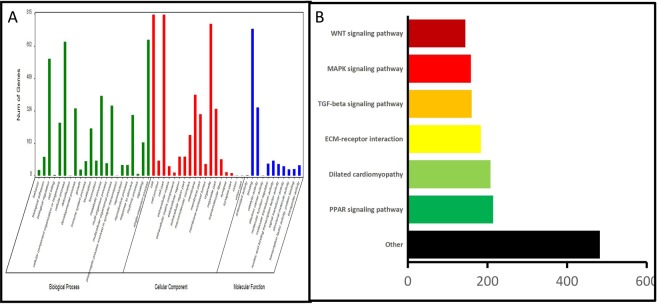
(**A**) GO Classification Statistical Map. The abscissa is the secondary classification description of GO, the ordinate is the number, and the three colors represent the three major classifications. (**B**) The top six signal path diagram. Abscissa is the number of enriched genes, and ordinates are signal pathways.

To further study the biological function of differentially expressed genes related to the development of secondary follicles in Inner Mongolia cashmere goats during fetal period, GO functional analysis and KEGG Pathway analysis were performed on 1548 differentially expressed genes. GO functional analysis includes three aspects: molecular function, cellular component and biological process involved in describing the attributes of genes (Fig. 3A). In biological processes, differential genes are mainly concentrated in biological regulation, cellular process and single-organism process. In cellular component, differential genes are mainly concentrated in cell, cell part, organelle process. In molecular function, differential genes are mainly concentrated in binding, catalytic activity process. It is presumed that the initiation of hair follicle development is mainly driven by the above eight factors.

A significant pathway list was obtained by KEGG analysis of 1548 differentially selected genes (Fig. 3B). The annotated Pathway is arranged from small to large according to p value and q-value. q-value is similar to FDR, both of which are a kind of correction for p value. The q-value of a hypothesis test is the minimum value of FDR, under which the hypothesis test can be considered significant. There are 23 significant pathways under the condition of p value and q value less than 0.05 standard threshold of difference, then the top 3 significant pathways are PPAR signaling pathway, dilated cardiomyopathy (DCM) and ECM-receptor interaction, if only consider p value, there are 46 significantly. In those pathway, TGFβ signaling pathway and MAPK signaling pathway were more studied in the development of skin hair follicle.

### Target gene prediction of miRNA

**Figure 4:**
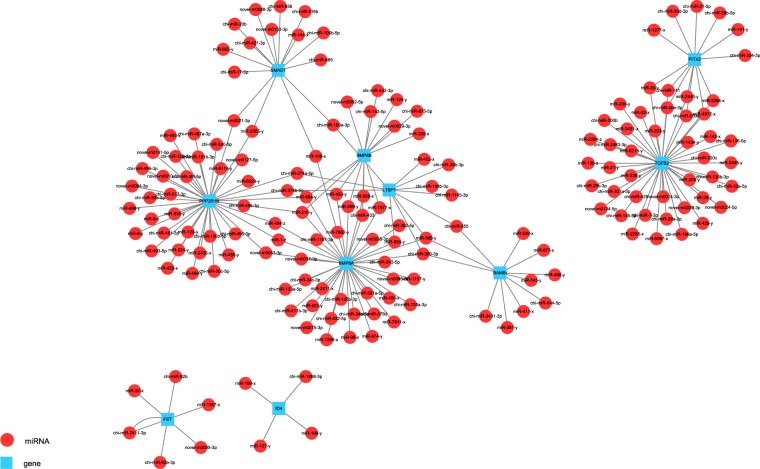
Interaction diagram between miRNA and target mRNA.

miRNA produces functions by interacting with target genes, Therefore, when studying the function of miRNA, it is important to predict target genes for miRNA. As the result shows (Table 3), there are 433 miRNA corresponding to 35545 target genes. In which there are common miRNA and target genes among different samples and one to many, many to one relationship between miRNA and target genes (Fig. 4). Hair follicle production is a process from nothing to existence, Therefore, removing the co-existing microRNAs and their target genes, obtaining the specific microRNAs and their target genes for each sample is the key to obtain the target genes related to hair follicle development.

**Table 3:** miRNA and target genes number.

sample name	miRNA number	target gene number	target number
Total	433	35545	907323
FP-45d	399	35330	826490
FP-55d	396	35335	816511
FP-65d	384	35245	799399
FP-75d	394	35305	819758
FP-95d	396	35317	824022
FP-115d	387	35282	809314
FP-135d	381	35228	801538

### Results of combined analysis of mRNA and miRNA

The general idea of the combined analysis of miRNA and mRNA is to find the target gene and target miRNA according to the negative correlation between miRNA expression and target gene expression given the targeted relationship between miRNA and mRNA. In this study, genes in TGF-β signaling pathway and MAPK signaling pathway, which have been studied extensively in cashmere goats, were compared to the gene data obtained following the combined analyses. Ten target genes and 195 target miRNAs were found in TGF-β signaling pathway related to the development of secondary hair follicles in Inner Mongolia cashmere goats. For the MAPK signaling pathway 26 target genes and 565 target miRNAs were found (Appendix A,B).

### Verification of sequencing results

**Figure 5:**
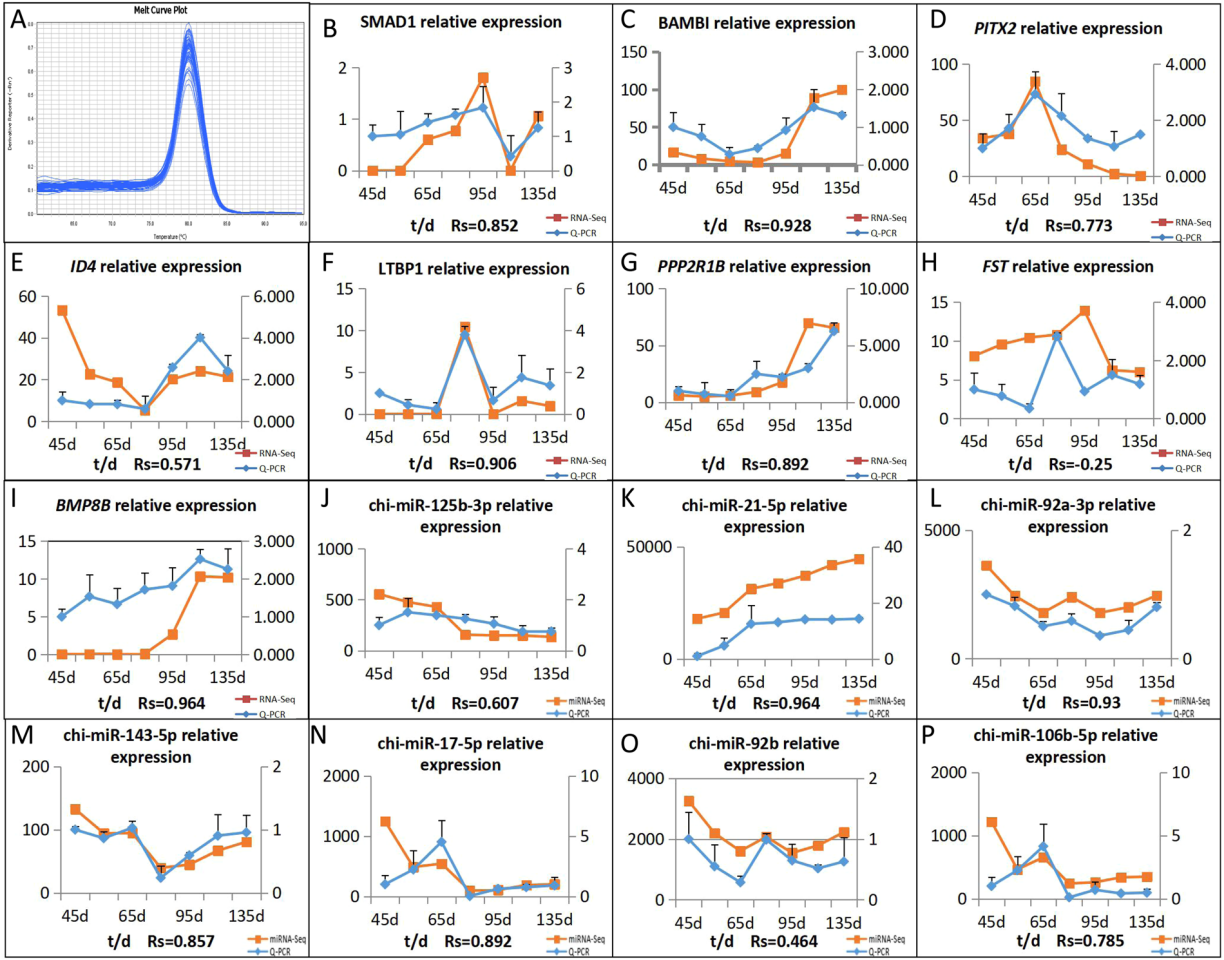
The expression quantity and expression trend of mRNA and miRNA in different periods. 0.8 < |Rs | <1 indicates a strong correlation; 0.6 < |Rs | <0.8 indicates a strong correlation; 0.4 < |Rs | <0.6 indicates a moderate correlation; 0.2 < |Rs | <0.4 indicates a weak correlation; 0 < |Rs | <0.2 indicates no correlation; The degree of proximity between |Rs| and 1 represents the degree of closeness and correlation between two variables.

We selected 8 genes and 7 miRNA which were more studied in the skin follicle for RT-qPCR verification (Fig. 5). The RT-qPCR amplification curve is a smooth S shape curve (Fig. 5A), all amplification initiates before the 30th cycle. This suggests that the amplification CT values are to be believed, dissolved no bimodal curve, for single piece, explain primers specificity is good. Overall, this analysis can show that the test using RT-qPCR system and good response procedures can be used for RT-qPCR to detect miRNA and its target genes in cashmere goat fetal period 45 days, 55 days, 65 days, 75 days, 95 days, 115 days and 135 days differentially expressed in the skin tissue. By qPCR quantitative analysis, we found that 7 genes (*BAMBI* (Fig. 5 C), *SMAD1* (Fig. 5B), *LTBP1* (Fig. 5F), *PPP2R1B* (Fig. 5G), *ID4* (Fig. 5E), *BMP8B*(Fig. 5I) and *PITX2* (Fig. 5D)) and 7 miRNA (*chi*-miR-17-5p (Fig. 5N), *chi*-miR-125b-3p (Fig. 5J), *chi*-miR-21-5p (Fig. 5K), *chi*-miR-143-5p (Fig. 5M), *chi*-miR-92a-3p (Fig. 5L), *chi*-miR-92b (Fig. 5O) and *chi*-miR-106b-5p (Fig. 5P)) RT-qPCR results on 45 days, 55 days, 65 days, 75 days, 95 days, 115 days, and 135 days expression in skin samples was consistent with the results of sequencing. And it is found that the target relationship between miRNA and mRNA is verified, *chi*-miR-17-5p and *chi*-miR-106b-5p began to interact with the gene SMAD1 at the age of 70 days and they have a negative regulatory effect on it. (Fig. 5B,N,P), *chi*-miR-21-5p is a negative regulator of *PITX2* gene from 80 days of age (Fig. 5D,K), *chi*-miR-143-5p also had negative regulation on gene *BMP8B* from 70 days old (Fig. 5I,M).

### Target identification by dual-luciferase reporter gene system

**Figure 6:**
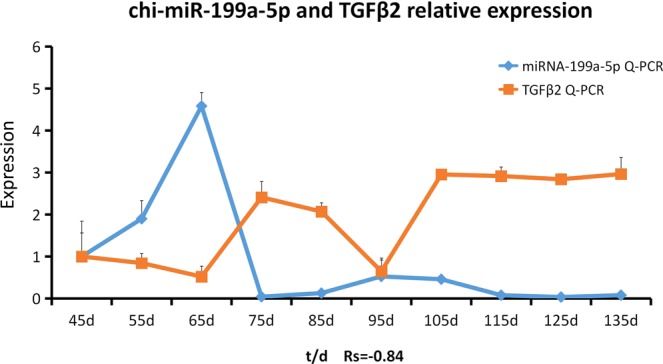
c*hi*-miR-199a-5p and *TGF-β2* relative expression. The correlation between the two variables is expressed by correlation coefficient Rs. The correlation coefficient is between ±0.50 and ±0.80, indicating a significant positive correlation between the two sets of data.

**Figure 7:**
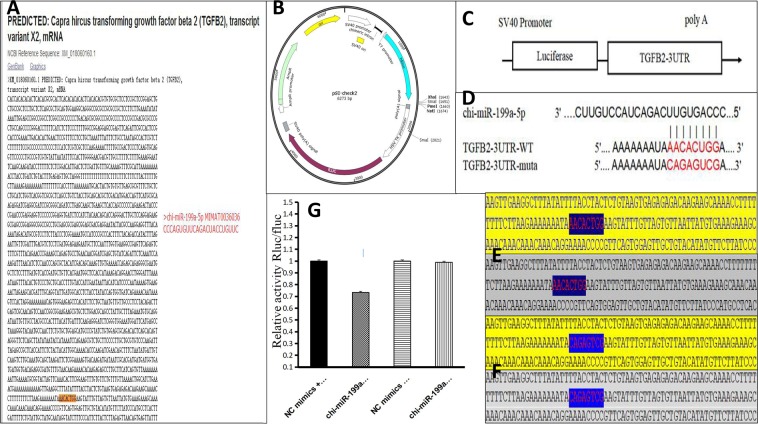
A Predicted binding sites of *chi*-miR-199a-5p and *TGF-β2*-3′UTR. BC psiCHECK-2 vector structure map and location of *TGF-β2*-3′UTR were cloned into psiCHECK-2 double luciferase reporter gene vector. The gene sequence was inserted into the XhoI and NotI digestion sites, and the expression was regulated by SV40 promoter. The vector contained Luc marker gene expression. D *Chi*-miR-199a-5p and *TGF-β2*-3′UTR target site binding sites and *TGF-β2* mutation sites. E *TGF-β2*-3′UTR wild type sequencing results. Grey is shown to be a sequence of clone targets, and yellow shows a predicted binding site. F *TGF-β2*-3 ‘UTR mutant sequencing results. Grey is shown to be a sequence of clone targets, and yellow shows a predicted binding site. G Detection of *chi*-miR-199a-5p and *TGF-β2*-3 ‘UTR interaction by dual luciferase reporter gene.

In order to further verify the regulatory relationship between microRNAs and genes, we further studied *TGF-β2* and *chi*-miR-199a-5p (Fig. 6). As the result shows, *TGFβ2* and *chi*-miR-199a-5p were expressed in 10 stages of cashmere goats fetal. R language was used to analyze the Spelman correlation coefficients of the two groups of data. The correlation coefficient between *TGFβ2* and *chi*-miR-199a-5p expression profile in fetal 45d-135d was −0.84, which showed a strong negative correlation. The expression of *TGFβ2* in the skin of gestational age 65 days–75 days was increased, and the expression of *TGFβ2* was also increased in 95 days–125 days. The targeting relationship between *TGF-β2* and *chi*-miR-199a-5p was preliminarily proved using RT-qPCR. The target was identified using the Dual-Luciferase Reporter Gene System. First, the gene sequence and vector sequence were analyzed (Fig. 7A). Amplification primer design based on *TGF-β2*−3′UTR sequence information. TGF-β2-3′UTR sequence was amplified from goat genomic DNA and cloned into psiCHECK-2 vector (Fig. 7B,C). Using the method of PCR mutation, the mutant vector was synthesized by designing mutated primers on the basis of wild type vector (Fig. 7D). psiCHECK-2-TGF-β2-3′UTR-wt (wild type) and psiCHECK-2-TGF-β2-3′UTR-mut (mutant) (Fig. 7E,F) sequencing results were aligned with the genebank reference sequence and were consistent with the expected results. It is here proved that TGF-β2-3′UTR has been successfully transferred into the psiCHECK-2 vector, and psiCHECK-2-TGF-β2-3′UTR-wt (wild type) and psiCHECK-2-TGF-β2-3′UTR-mut (mutant) have been constructed successfully. miRNA mimics were co-transformed with the constructed reporter gene vector, and the interaction between miRNA and the target gene was confirmed by down-regulation of the relative fluorescence value of the reporter gene. The results showed that the luciferase expression of *TGF-β2*-3′UTR was significantly down-regulated by *chi*-miR-199a-5p, indicating that there was a binding effect between the two. This downregulation disappeared after mutation, indicating that the mutation was successful (Fig. 7G).

### Function of miRNA-199a-5p in fibroblasts of Inner Mongolia cashmere goats

**Figure 8:**
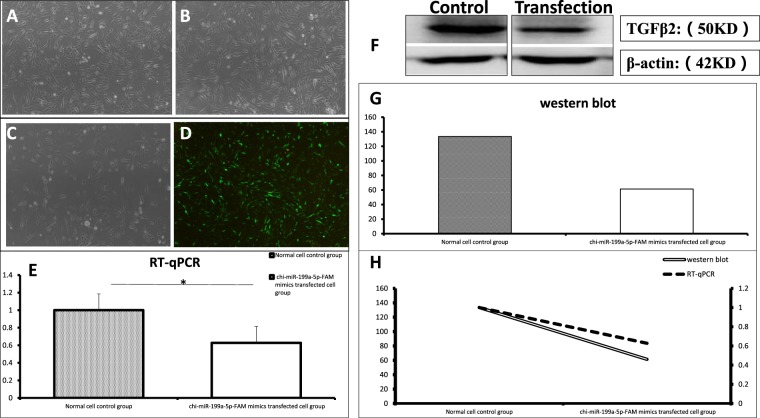
A,B Fibroblast culture. C Transfection of empty carrier cells. D Cells transfected with miR-199a-5p-FAM mimics vector. E The relative expression of *TGFβ2* gene in defferent treatments in Inner Mongolia cashmere goat fibroblasts cell. F The expression of *TGF-β2* protein. G The relative expression of *TGF-β2* protein in defferent treatments in Inner Mongolia cashmere goat fibroblasts cell. H The expression trend of *TGF-β2* mRNA and protein after *chi*-miR199a-5 p-FAM mimics transfection.

Culture of Inner Mongolia goat fibroblasts (Fig. 8A,B) and transfecting Plasmid vectors into cells. As the result show that no green fluorescence was observed in cells transfected with empty vector plasmids (Fig. 8C) and miR-199a-5p-FAM mimics transfected fibroblasts showed strong green fluorescence with uniform distribution (Fig. 8D). RT-qPCR results show that the expression level of target gene (*TGFβ2*) mRNA in cells (Fig. 8E) decreased significantly after transfection of miR-199a-5p-FAM mimics (p < 0.05). Western blot results showed that the expression of target gene (*TGFβ2*) was significantly lower in microRNAs transfected cells than in the control group (Fig. 8F). The amount of protein expression was indicated by the gray value of protein bands. The higher the gray value after the internal parameters correction, the more the target protein expression. The gray-level values of TGFβ2 protein in the control group and the *chi*-miR-199a-5p-FAM mimics transfected cell group were 133.55 and 61.36, respectively, after correction of internal reference (Fig. 8G).Compared with the normal control group, both *TGFβ2* mRNA and protein levels were significantly decreased in the chi-miR199a-5p-FAM mimics transfected fibroblast group. mRNA expression decreased by 37% and protein level decreased by 54%.The mRNA and protein levels of the *chi*-miR-199a-5p-FAM mimics transfected fibroblast group were significantly lower than those of the normal cell control group (Fig. 8H). mRNA expression decreased by 37% and protein level decreased by 54%. These results fully illustrate the regulatory mechanism of miR-199a-5p on the negative regulation of *TGF-β2* gene during hair follicle development.

## Discussion

Transcriptome sequencing is based on the second generation sequencing technology^17^. The transcriptional set of genes refers to the sum of all RNA that can be transcribed in one cell or group of cells under the same environment or physiological conditions, including mRNA and various non-coding RNAs (lncRNA, miRNA)^18^. High throughput sequencing is used to obtain almost all transcriptional sequence information of a specific tissue or organ of a species during a certain state. Transcriptome sequencing can reflect the gene expression of the species at a specific time and in a specific tissue^19^. GO function analysis and KEGG pathway analysis screened two pathways, TGF-beta signaling pathway and MAPK signaling pathway^20,21^, which have been extensively studied in cashmere goat. These pathways have also been reported to be important pathways associated with hair follicle development. We finally selected 8 genes and 7 miRNAs to verify the sequencing results. We found that expression of 7 genes *(BAMBI*, *SMAD1*, *LTBP1*, *PPP2R1B*, *ID4*, *BMP8B* and *PITX2)* and 7 miRNA (*chi*-miR-17-5p, *chi*-miR-125b-3p, *chi*-miR-21-5p, *chi*-miR-143-5p, *chi*-miR-92a-3p, *chi*-miR-92b and *chi*-miR-106b-5p) RT-qPCR in skin samples representing 45 days, 55 days, 65 days, 75 days, 95 days, 115 days, and 135 days into the fetal development period was consistent with the results of sequencing. The trends in the expression of *FST* were not completely consistent with the sequencing results. In conclusion, the results of sequencing are reliable.

The key gene that co-exists in TGF-β and MAPK pathways is *TGFβ2*. By association analysis, 41 possible target miRNAs were obtained, and 16 of them were known to exist in goat species. We chose *chi*-miR-199a-5p for subsequent validation. The TGF-β signaling pathway is a multifunctional cytokine pathway widely present in various organisms. It belongs to the transforming growth factor superfamily. TGF-β is named according to the fact that this cytokine can transform the phenotype of normal fibroblasts. That is, in the presence of epidermal growth factor (EGF), the adherent growth of fibroblasts is changed and the phenotypes of normal fibroblasts are transformed. The genes of three isomers of TGFβ (β1, β2, β3)^22^ are highly conserved in different species in mammals. In the process of TGFβ signal transduction, activated TGFβ binds to receptor on cell membrane and further transduction of signal. Three subtypes of type I receptor and type III receptor. The TGFβ signaling pathway includes three subtypes of type I, type II and type III receptor^23^. *TGFβ1*, *TGFβ2* and *TGFβ3* are very similar in most biological functions, but they may differ greatly in some aspects. *TGFβ1* is closely related to the occurrence and development of many diseases, such as inflammation and trauma, and it participates in various pathophysiological processes in the body, especially for the occurrence and development of tumor^9^; *TGFβ*2 plays an important role in cell proliferation, differentiation, embryonic development, tumor inhibition and metastasis and diffusion. *TGFβ2* can induce the formation of mesoderm during embryonic development and promote the formation of hair follicles and other skin auxiliary structures^24^; *TGFβ1-*3 can regulate bone formation and affect adult bone regeneration. *TGFβ* family members play an important role in the development of hair follicles^25^. *TGFβ1* plays a facilitating role in the transition of hair follicles from growth to catagen^26^. Mice with knockout of *TGFβ1* have prolonged hair follicle catagen^27^. Overexpression of *TGFβ1* inhibits hair follicle development in mice^28^. *TGFβ2* can induce hair follicles to transition from rest to growth^21^. However, it is not clear how to regulate the development of hair follicles in Inner Mongolia cashmere goats during the fetal period.

In addition to existing in the pathway of the signal molecules, miRNA in hair follicles of the skin also play an important role in the process of development^29^. The first gene found to be highly expressed in the skin hair follicle is miR-203. miR-203 expression quantity in normal skin is 100 times higher than that of other organs. It is considered to be the skin-specific miRNA and thought to be closely related to hair follicle development. The members of the miR-200 family, miR-196a and miR-125b, have long been considered closely related to hair follicle development and to participate in the regulation of hair follicle development^12^. The expression of miR-200b was significantly different in all stages of hair follicle development, and it was significantly higher during the middle and late stages of hair follicle development than during the early stage of hair follicle development. It is assumed that the expression level of miR-200b plays an important role in the development of hair follicles^30^; miR-199a-5p can inhibit the proliferation of keloid fibroblasts, suggesting that miR-199a-5p may be involved in the regulation of keloid fibroblast proliferation-related genes^31^. By constructing a small molecular library of 70 day cashmere goat fetal skin and comparing it to miRNA from mouse fetal skin and hair follicles, it was found that miR-199a, miR199a*, miR-214a and miR-127 were expressed in cashmere goat and mouse skin, and there was more than three times as much expression of miR-199a, miR199a* and miR-214 in mouse hair follicles than in mouse skin. It is assumed that they are directly related to the development of hair follicles^32^.

The results of RT-qPCR showed that the expression of *TGF-β2* and *chi*-miR-199a-5p was negatively correlated with the expression of 45–135 days in the fetal stage. The expression of *TGF-β2* in the skin during gestational age 65–75 days was increased, and the expression of *TGF-β2* was also increased in samples representing 95–125 days. In mice with *TGF-β2* deficiency, the delayed development of hair follicles and the decrease in the number of hair follicles indicated that *TGF-β2* showed the characteristic of stimulating the development of hair follicles^28^; At the gestational age of 65 days, the primary hair follicle buds were observed in the Inner Mongolia cashmere goat. At 65–75 days, the primary body of the secondary hair follicle was observed in all parts of the fetus, and the secondary hair follicle began to appear. The density of secondary hair follicles increased at gestational age (95–105 days) and reached its maximum at 105 days. In conclusion, *TGF-β2* may play a promoting role in the development of hair follicles. However, *chi*-miRNA-199a-5p negatively regulates *TGF-β2*, so the role of *TGF-β2* in the development of hair follicles cannot be underestimated.

When the expression of miRNA is inversely proportional to the expression of predicted target gene, the targeting effect between miRNA and target gene can be proved, but there is a large false positive in this method. Therefore, we the selected Dual-Luciferase Reporter Gene System to detect the regulatory effect of *chi*-miR-199a-5p on target gene *TGF-β2* and verify whether it regulates the expression of *TGF-β2*^33^. In this test, psiCHECK™-2 vector contains a second reporter gene-firefly luciferase, was designed to end point cracking detection. Its function is to normalize the expression of the Renilla luciferase and obtain strong and repeatable data. The purpose of this experiment was to verify the interaction between molecules. Cells are only the mediators. It is not necessary to select the target cells for the experiment. Therefore, 293 T cells with high transfection efficiency and easy transfection are selected as the experimental cells. The results of the Dual-Luciferase Reporter Gene System showed that chi-miR-199a-5p directly regulated the expression of *TGF-β2* and inhibited the expression of *TGF-β2*.

The Dual-Luciferase Reporter Gene System only indicates that there is a binding between the two, which does not indicate whether miR-199a-5p prevents *TGF-β2* expression at the mRNA level or at the post-transcriptional translation level. Therefore, we further investigated how miR-199a-5p regulates the expression of the *TGF-β2* gene. We transfected *chi*-miR199a-5 p-FAM mimics into *in vitro* cultured Inner Mongolia cashmere goat fibroblasts. After transfection, the cells were harvested to extract total RNA and protein. The mRNA and protein expression levels of *TGF-β2* in fibroblasts were detected by RT-qPCR and western blot. By transfecting fibroblasts cultured *in vitro*, it was found that *chi*-miR-199a-5 p-FAM mimics was transfected into fibroblasts for 72 h, and the mRNA and protein levels were significantly decreased compared with the normal cell control group, which was verified that *chi*-miR-199a-5p inhibits *TGF-β2* expression at both mRNA and protein translation levels in fibroblasts. At the same time, it was again proved that the *TGF-β2* gene is a target gene of *chi*-miR-199a-5p. In summary, this provides a theoretical basis for further studying the regulation of hair follicle development by *chi*-miR-199a-5p and *TGF-β2*.

## Materials and Methods

### Tissue sample collection

The study samples were collected from an Inner Mongolian Arbas white cashmere goats breeding farm, All animal experiments were performed in accordance with the ‘Guidelines for Experimental Animals’ of the Ministry of Science and Technology (Beijing, China). All surgery was performed according to recommendations proposed by the European Commission (1997), and was approved by experimental animal ethics committee of Inner Mongolia Agricultural University. A total of 7 samples were collected 45 days, 55 days, 65 days, 75 days, 95 days, 115 days, and 135 days into the gestation period from 3 fetuses at each time period. The skin of the fetus was collected, washed with DEPC water, and snap frozen in liquid nitrogen. Samples were taken back to the laboratory and stored in a − 80 °C freezer for later use.

### Total RNA extraction, library construction and sequencing

The mRNA and miRNA sequencing of 9 skin samples representing the fetal period of the cashmere goats (45 days, 55 days and 65 days, 3 samples for biological repetition in each period) was completed in the early laboratory. To save on costs, the study sequenced 12 samples from the side skin of the fetus at 75 days, 95 days, 115 days and 135 days of cashmere goats. Using the Trizol reagent kit (Takara, Japan) method for extraction of total RNA, 12 samples of total RNA were placed in −80 °C refrigerated storage. mRNA and small RNA libraries were established. Agilent 2100 (Agilent, CA, USA) was used for library quality inspection. Illumina HiSeq TM 4000 (Illumina, CA, USA) was sequenced. mRNA and small RNA library construction and sequencing were performed by Gene Denovo in Guangzhou. In order to ensure data quality, quality control should be carried out on the original data before analysis and data filtering should be adopted to reduce data noise. We filter clean reads more strictly and obtain high quality clean reads for subsequent bioinformatics analysis.

### Differential analysis

In order to make the expression abundance of different sequencing samples comparable, gene expression was calculated using FPKM (fragments per kilobase of transcript per million mapped reads). The calculation formula is as follows:

$$FPKM=\frac{exo{n}_{reads}\times {10}^{9}}{uniqu{e}_{reads}\times gen{e}_{length}}$$

The normalized value of each miRNA expression is expressed with TPM (tags per million), and the formula is:

$$TPM=T\ast \frac{{10}^{6}}{N}$$

(T denotes the tags of miRNA, N denotes the tags of the total miRNA).

The original data from the two sequencing results were here reanalyzed using bioinformatics. We used the R-based software package edgeR (http://www.bioconductor.org/packages/release/bioc/html/edger.html) to determine whether there was a significant difference in the amount of expression between the two groups. For the mRNA, we used a false discovery rate (FDR) and log2(FC) (FC: fold change) was used to screen differentially expressed genes. The screening conditions were FDR < 0.05 and |log2FC | > 1. The screening criteria for miRNA differences were as follows: the expression level changed more than 2 times and the *p* value was less than 0.05.

### Gene screening

This study is designed to discover differentially expressed genes related to secondary follicle development and its corresponding miRNA. In the preliminary study on the structure and morphogenesis of hair follicles of cashmere goats, it was found that the skin formed a complete epidermis. The hair follicle had not occurred at the 45 to 55 day of the embryonic period. At 55 to 65 days, primary hair follicles began to develop in all parts of the fetus. At 65 to 75 days, secondary hair follicle primordial bodies can be observed at all parts of the fetus. Secondary hair follicle begins to occur and grows out of the epidermis near the primary hair follicle^34^. According to previous studies, it was inferred that there were no significant differences among the genes in the three stages at 45 days, 55 days and 65 days, but after 75 days, the genes and their corresponding miRNA were different that are likely to be associated with secondary follicle development. Therefore, we use the union of the first 3 time points at 45 days, 55 days and 65 days of differential genes at stage A and the last 4 time points at 75 days, 95 days, and 135 days of differential genes at stage B to make the Venn diagram (Fig. 3). Stage B removes the common part from stage A, and the rest may be genes related to secondary follicle development (http://www.biocloud.net/).

### GO function analysis and KEGG pathway analysis

GO function analysis and KEGG Pathway analyses were carried out for the selected genes related to secondary follicle development. On the one hand, functional annotations were made for them; on the other hand, we hope to find out the main relevant functions of these candidate genes. GO function analysis is a standardized system of gene function annotation. First, differentially expressed genes are mapped to the related GO database (http://www.geneontology.org/) of each term, and the number of genes in each term is calculated. A GO function gene list and the number of the statistics are also determined. In total, GO has 3 ontologies, which respectively describe the molecular function, cellular component and biological process of genes. Pathway-based analysis is helpful to further understand the biological function of genes. KEGG is the main public database on Pathway^4^. GO and KEGG Pathway analysis were performed using the OmicShare tools, a free online platform for data analysis (www.omicshare.com/tools).

### miRNA target gene prediction

In animals, miRNA binds to the target gene mRNA 3′UTR in an incomplete complementary pairing manner. According to this characteristic, in order to increase accuracy, we made RNAhybrid (v2.1.2) + svm_light (v6.01), Miranda (v3.3 a) and TargetScan (7.0) Version 3 methods to existing miRNA target genes prediction, then took 3 ways to get the intersection as the miRNA target genes to predict the results of the forecasting results of target genes.

### Combined analysis of mRNA-miRNA

The differentially expressed genes and differentially expressed miRNA with target relationships were selected and the correlation coefficient was obtained using the expression quantity. Due to the negative regulation of target mRNA by miRNA, we listed the miRNA-target gene with a Spielman rank correlation coefficient of less than −0.5. One possible negatively regulated miRNA-target gene pair was found.

### Validation of sequencing data

We finally selected 8 genes and 7 miRNAs to verify the sequencing results by RT-qPCR. The primer was designed and synthesized by Wuhan Biofavor Bioceth Service Co., LTD(Hu bei). Primer sequences are shown in Table 4 and Table 5.

**Table 4:** mRNA primer sequence.

Name	Primer	Sequence	Size
b-actin	Forward	5′-GGCATTCACGAAACTACCTT-3′	263 bp
	Reverse	5′-TGCTTGCTGATCCACATCT -3′	
SMAD1	Forward	5′-AGAAGGCCGTTGATGCTTTG -3′	231 bp
	Reverse	5′-ATTCCAACGGCTTCAGTTCG -3′	
BAMBI	Forward	5′-GCGTGGCTACTGGTTACATG -3′	189 bp
	Reverse	5′-GACAGCACTCCAAAGAAGGC-3′	
PITX2	Forward	5′- GACCAACCTAACGGAAGCAC -3′	225 bp
	Reverse	5′- AAAGGGAAGCTCTTGGTGGA -3′	
LTBP1	Forward	5′-GCAAAGCCTGTGAGACAACA -3′	201 bp
	Reverse	5′-AGAGTCCCACTGAAGGCTTC -3′	
BMP8B	Forward	5′- TCGTGGTCACCTTTTTCAG -3′	268 bp
	Reverse	5′- CACTCCCCTTCACAGTAATA -3′	
ID4	Forward	5′- CCCGCCCAACAAGAAAGTCA -3′	232 bp
	Reverse	5′- GAGAATGCTGTCGCCCTGCT -3′	
PPP2R1B	Forward	5′- GCCTTTCAGAACCTACTCAA -3′	287 bp
	Reverse	5′- CGAACTTCAGGACACTCAT -3′	
FST	Forward	5′- GCCAGATGTAAAGAGCAGCC -3′	227 bp
	Reverse	5′- GCTTTTCTCAGGTGACAGGC -3′

**Table 5:** miRNA primer sequence.

Name	Primer	Sequence
U6	Forward	5′- CGCTTCGGCAGCACATATAC -3′
	Reverse	5′- AAATATGGAACGCTTCACGA -3′
*chi*-miR-106b-5p	Loop primer	5′-GTCGTATCCAGTGCAGGGTCCGAGGTATTCGCACTGGATACGAC ATCTGCAC -3′
	F primer	5′-TGCGCTAAAGTGCTGACAGTG -3′
*chi*-miR-17-5p	Loop primer	5′-GTCGTATCCAGTGCAGGGTCCGAGGTATTCGCACTGGATACGAC ACTACCTG -3′
	F primer	5′-TGCGC CAAAGTGCTTACAGTGCAG -3′
*chi*-miR-21-5p	Loop primer	5′-GTCGTATCCAGTGCAGGGTCCGAGGTATTCGCACTGGATACGAC GTCAACAT -3′
	F primer	5′-TGCGC TAGCTTATCAGACTGATG-3′
*chi*-miR-125b-3p	Loop primer	5′-GTCGTATCCAGTGCAGGGTCCGAGGTATTCGCACTGGATACGAC GGTCCCAA -3′
	F primer	5′-TGCGC ACAAGTCAGGCTCTTG -3′
*chi*-miR-143-5p	Loop primer	5′-GTCGTATCCAGTGCAGGGTCCGAGGTATTCGCACTGGATACGAC CCAGAGAT -3′
*chi*-miR-92a-3p	Loop primer	5′-GTCGTATCCAGTGCAGGGTCCGAGGTATTCGCACTGGATACGAC ACAGGCCG -3′
	F primer	5′-TGCGC TATTGCACTTGTCCCGG -3′
*chi*-miR-92b	Loop primer	5′-GTCGTATCCAGTGCAGGGTCCGAGGTATTCGCACTGGATACGAC ACAGGCCG -3′
	F primer	5′-TGCGC TATTGCACTTGTCCCGG -3′
	F primer	5′-TGCGCGGTGCAGTGCTGCATC -3′

### Target identification using a dual-luciferase reporter gene system

The key TGF-β and MAPK signaling pathways related to the development of secondary follicles were predicted using a series of previous bioinformatics analyses. One key gene that co-exists in two of these pathways is *TGF-β*. By association analysis, 41 possible target miRNA were obtained, and 16 of them were already known to exist in goat species—the miRNAs beginning with chi are already present in goat species. We chose chi-miR-199a-5p for subsequent validation. At present, most laboratories use RT-qPCR to identify target gene candidates. When the amount of miRNA and predicted target gene expression is inversely proportional, it can be proved that there is a targeting effect between the two^35^. However, this method cannot accurately identify the location of the target. At present, the most commonly used target identification method is Dual-Luciferase Reporter Gene System^36^. The principle of double luciferase reporter gene is that miRNA works mainly by acting on the 3′UTR of the target gene. The target gene 3′UTR sequence can be constructed into the 3′UTR region of Renilla luciferase in the vector. miRNA mimics were co-transfected into cells with the constructed reporter gene vector. When the miRNA binds to the inserted sequence in the vector, it will inhibit the translation of fluorescent protein by binding to the inserted sequence, decreasing the level of fluorescence. In the process of measurement, firefly luciferase activity was first measured, and then the reaction was quenched, and the Renilla luciferase was initiated. The second measurement was called a Dual-Luciferase Reporter Gene System experiment^37^. The interaction between miRNA and target gene was confirmed by down-regulation of relative fluorescence of the reporter gene. The site of interaction between miRNA and the target gene 3′UTR was further determined by site-directed mutation.

### Function of miRNA-199a-5p in fibroblasts of Inner Mongolia cashmere goats

Inner Mongolia cashmere goat fibroblasts are preserved in the Key Laboratory of Animal Genetics, Breeding and Reproduction, Inner Mongolia Autonomous Region. *chi*-miR-199a-5p-FAM mimics was designed and synthesized by Hanbio biolotechnology Co., Ltd. Transfection according to the instructions for use of the miRNA product, and detection was performed 24 to 72 hours after the completion of transfection. The optimal detection time is related to the cell type and the miRNA being studied. Extraction of RNA from transfected fibroblasts according to TRIzol method (Bi yun tian, China).The expression level of target gene (*TGF-β2*) mRNA after chi-miR-199a-5p-FAM mimics transfected cells was detected by RT-qPCR according to the instructions of SYBR green I kit (western biotechnology). Primers were designed and synthesized by the Western Biomedical technology. *TGF-β2* forward primer: 5′-AGTCGCAACAGTCCAACCG- 3′, *TGFβ2* reverse primer : 5′-GGCATTGTACCCTTTAGGCTC-3′. β-actin as an internal reference,β-actin forward primer: 5′ -TCCTGCGTCTGGACCTGG -3′, β-actin reverse primer: 5′-CCTTGATGTCACGGACGATTC-3′. The 2^–ΔΔCt^ method was applied to conduct a relative quantitative analysis of the teal-time PCR data. Finally, variance analysis of the data was conducted using SAS 9.0 software with a level significance at p < 0.05.Western blot was used to detect *TGF-β2* in the fibroblasts were transfected with miR-199a-5p. First, the collected cells were added into the protein lysis solution (bi yun tian, China), the total protein was extracted according to the instructions, and the protein concentration was measured by BCA method. Then, 10% separating gel and 5% stacking gel were prepared for SDS-PAGE (polyacrylamide gel electrophoresis, PAGE). Finally, imaging scans. *TGF-β2* Primary antibodies (rabbit source) were purchased from biorbyt (UK), Internal Reference β-actin Primary antibodies (rabbit source) from abcam (US), and Secondary antibodies from sigma (US).

## Supplementary information

Appendix A and B.
