# Supplementary material for: Inner Mongolian Cashmere Goat Secondary Follicle Development Regulation Research Based on mRNA-miRNA Co-analysis
Source: Sci Rep. 2020 Mar 11;10:4519. doi: 10.1038/s41598-020-60351-5 (PMC7066195; doi:10.1038/s41598-020-60351-5)
Supplement: Supplementary file 1 — Appendix A and B. [file 41598_2020_60351_MOESM1_ESM.docx]

**Inner Mongolian Cashmere Goat Secondary Follicle Development Regulation Research Based on mRNA-miRNA Co-analysis**

Wenjing Han^1,*^,Feng Yang^1,*^, Zhihong Wu^1^，Fuqiang Guo^1^，Junjie Zhang^1^ , Erhan Hai ^1^,Fangzheng Shang^1^,Rui Su^1^, Ruijun Wang^1,2^, Zhiying Wang^1^ ,Zhihong Liu^1^, Yanhong Zhao^1^, Zhixin Wang^1^,Yanjun Zhang^1,3,#^ and Jinquan Li^1,4,#^

^1^ College of Animal Science, Inner Mongolia Agricultural University, Hohhot, Inner Mongolia Autonomous Region 010018, China

^2^ Key Laboratory of Animal Genetics, Breeding and Reproduction in Inner Mongolia Autonomous Region, Hohhot, Inner Mongolia Autonomous Region 010018, China

^3^ Engineering Research Center for Goat Genetics and Breeding, Inner Mongolia Autonomous Region, Hohhot, Inner Mongolia Autonomous Region 010018, China

^4^ Key Laboratory of Mutton Sheep Genetics and Breeding, Ministry of Agriculture, Hohhot, Inner Mongolia Autonomous Region 010018, China

*Co first author

#Corresponding author:

Yanjun Zhang

Tel: +86-13190601108, Fax: + 4309178, E-mail: [imauzyj@163.com](mailto:imauzyj@163.com);

Jinquan Li

Tel: +86-13947131345, Fax: +4309170, E-mail: lijinquan_nd@126.com;

Appendix A

| **TGF-beta signaling pathway（**10 target genes and 195 target miRNAs**）** | | | |
| --- | --- | --- | --- |
| ID | GeneID | rho | Symbol |
| miR-1357-x | XM_005694718.3 | -0.612372436 | FST |
| novel-m0005-3p | XM_005694718.3 | -0.630656224 | FST |
| chi-miR-92a-3p | XM_005694718.3 | -0.642857143 | FST |
| chi-miR-92b | XM_005694718.3 | -0.642857143 | FST |
| chi-miR-2411-3p | XM_005694718.3 | -0.518874522 | FST |
| miR-32-x | XM_005694718.3 | -0.607142857 | FST |
| chi-miR-2411-3p | XM_005694718.3 | -0.518874522 | FST |
| miR-127-y | XM_018039249.1 | -0.535714286 | ID4 |
| miR-199-x | XM_018039249.1 | -0.535714286 | ID4 |
| chi-miR-1468-3p | XM_018039249.1 | -0.612637475 | ID4 |
| miR-199-y | XM_018039249.1 | -0.571428571 | ID4 |
| chi-miR-369-3p | XM_018042149.1 | -0.928571429 | BMP8A |
| chi-miR-133a-3p | XM_018042149.1 | -0.928571429 | BMP8A |
| chi-miR-362-5p | XM_018042149.1 | -0.928571429 | BMP8A |
| chi-miR-432-5p | XM_018042149.1 | -0.75 | BMP8A |
| chi-miR-125b-3p | XM_018042149.1 | -0.857142857 | BMP8A |
| chi-miR-433 | XM_018042149.1 | -0.75 | BMP8A |
| chi-miR-1197-3p | XM_018042149.1 | -0.857142857 | BMP8A |
| chi-miR-376d | XM_018042149.1 | -0.857142857 | BMP8A |
| chi-miR-301a-5p | XM_018042149.1 | -0.857142857 | BMP8A |
| chi-miR-34b-3p | XM_018042149.1 | -0.846881215 | BMP8A |
| chi-miR-411b-3p | XM_018042149.1 | -0.714285714 | BMP8A |
| chi-miR-34c-3p | XM_018042149.1 | -0.821428571 | BMP8A |
| miR-99-x | XM_018042149.1 | -0.607142857 | BMP8A |
| miR-450-x | XM_018042149.1 | -0.928571429 | BMP8A |
| miR-299-y | XM_018042149.1 | -0.857142857 | BMP8A |
| miR-484-z | XM_018042149.1 | -0.785714286 | BMP8A |
| miR-106-x | XM_018042149.1 | -0.607142857 | BMP8A |
| miR-503-y | XM_018042149.1 | -0.821428571 | BMP8A |
| miR-7977-x | XM_018042149.1 | -0.642857143 | BMP8A |
| miR-369-y | XM_018042149.1 | -0.964285714 | BMP8A |
| miR-1298-x | XM_018042149.1 | -0.821428571 | BMP8A |
| miR-433-y | XM_018042149.1 | -0.892857143 | BMP8A |
| miR-655-y | XM_018042149.1 | -0.88949918 | BMP8A |
| miR-874-y | XM_018042149.1 | -0.928571429 | BMP8A |
| miR-7862-y | XM_018042149.1 | -0.821428571 | BMP8A |
| miR-1197-y | XM_018042149.1 | -0.714285714 | BMP8A |
| miR-216-y | XM_018042149.1 | -0.785714286 | BMP8A |
| miR-664-y | XM_018042149.1 | -0.788110406 | BMP8A |
| miR-7911-x | XM_018042149.1 | -0.669893845 | BMP8A |
| novel-m0075-3p | XM_018042149.1 | -0.788110406 | BMP8A |
| novel-m0095-3p | XM_018042149.1 | -0.788110406 | BMP8A |
| novel-m0035-3p | XM_018042149.1 | -0.579066024 | BMP8A |
| novel-m0037-3p | XM_018042149.1 | -0.579066024 | BMP8A |
| novel-m0053-3p | XM_018042149.1 | -0.579066024 | BMP8A |
| chi-miR-133a-5p | XM_018042149.1 | -0.857142857 | BMP8A |
| miR-1-z | XM_018042149.1 | -0.857142857 | BMP8A |
| chi-miR-543-5p | XM_018042149.1 | -0.704186851 | BMP8A |
| miR-2411-x | XM_018042149.1 | -0.669893845 | BMP8A |
| miR-508-x | XM_018042149.1 | -0.778311782 | BMP8A |
| chi-miR-433 | XM_018042197.1 | -0.571428571 | BMP8B |
| chi-miR-432-3p | XM_018042197.1 | -0.535714286 | BMP8B |
| chi-miR-873-5p | XM_018042197.1 | -0.857142857 | BMP8B |
| miR-299-y | XM_018042197.1 | -0.714285714 | BMP8B |
| miR-335-x | XM_018042197.1 | -0.857142857 | BMP8B |
| miR-7977-x | XM_018042197.1 | -0.535714286 | BMP8B |
| miR-433-y | XM_018042197.1 | -0.785714286 | BMP8B |
| miR-216-y | XM_018042197.1 | -0.678571429 | BMP8B |
| miR-664-y | XM_018042197.1 | -0.906326967 | BMP8B |
| novel-m0029-3p | XM_018042197.1 | -0.821428571 | BMP8B |
| novel-m0052-5p | XM_018042197.1 | -0.821428571 | BMP8B |
| miR-508-x | XM_018042197.1 | -0.555936987 | BMP8B |
| chi-miR-143-5p | XM_018042197.1 | -0.642857143 | BMP8B |
| miR-124-y | XM_018042197.1 | -0.571428571 | BMP8B |
| chi-miR-21-5p | XM_018049158.1 | -0.857142857 | PITX2 |
| chi-miR-200a | XM_018049158.1 | -0.857142857 | PITX2 |
| chi-miR-141 | XM_018049158.1 | -0.75 | PITX2 |
| chi-miR-30e-3p | XM_018049158.1 | -0.857142857 | PITX2 |
| miR-2284-x | XM_018049158.1 | -0.571428571 | PITX2 |
| miR-141-y | XM_018049158.1 | -0.857142857 | PITX2 |
| miR-1277-x | XM_018049158.1 | -0.678571429 | PITX2 |
| miR-2440-y | XM_018049158.1 | -1 | PITX2 |
| miR-4872-x | XM_018049158.1 | -0.668153105 | PITX2 |
| chi-miR-30d-3p | XM_018049158.1 | -0.785714286 | PITX2 |
| chi-miR-324-3p | XM_018049158.1 | -0.75 | PITX2 |
| chi-miR-29b-5p | XM_018049158.1 | -0.95499371 | PITX2 |
| miR-30-y | XM_018049158.1 | -0.75 | PITX2 |
| chi-miR-374a-5p | XM_018055105.1 | -0.591082805 | LTBP1 |
| chi-miR-369-3p | XM_018055105.1 | -0.571380045 | LTBP1 |
| chi-miR-148b-3p | XM_018055105.1 | -0.669893845 | LTBP1 |
| chi-miR-655 | XM_018055105.1 | -0.748704886 | LTBP1 |
| chi-miR-374b-5p | XM_018055105.1 | -0.512271764 | LTBP1 |
| chi-miR-1185-3p | XM_018055105.1 | -0.571380045 | LTBP1 |
| chi-miR-26b-3p | XM_018055105.1 | -0.807813166 | LTBP1 |
| chi-miR-190a-3p | XM_018055105.1 | -0.866921447 | LTBP1 |
| miR-452-x | XM_018055105.1 | -0.571380045 | LTBP1 |
| miR-369-y | XM_018055105.1 | -0.571380045 | LTBP1 |
| miR-655-y | XM_018055105.1 | -0.858753861 | LTBP1 |
| chi-miR-369-3p | XM_018057206.1 | -0.571428571 | BAMBI |
| chi-miR-655 | XM_018057206.1 | -0.571428571 | BAMBI |
| chi-miR-544-5p | XM_018057206.1 | -0.678571429 | BAMBI |
| miR-381-y | XM_018057206.1 | -0.642857143 | BAMBI |
| miR-369-y | XM_018057206.1 | -0.678571429 | BAMBI |
| miR-877-x | XM_018057206.1 | -0.607142857 | BAMBI |
| miR-539-x | XM_018057206.1 | -0.678571429 | BAMBI |
| miR-495-y | XM_018057206.1 | -0.571428571 | BAMBI |
| miR-543-y | XM_018057206.1 | -0.571428571 | BAMBI |
| miR-411-x | XM_018057206.1 | -0.678571429 | BAMBI |
| chi-miR-3431-3p | XM_018057206.1 | -0.964285714 | BAMBI |
| chi-miR-374a-5p | XM_018059799.1 | -0.75 | PPP2R1B |
| chi-miR-30c-5p | XM_018059799.1 | -0.821428571 | PPP2R1B |
| chi-miR-493-5p | XM_018059799.1 | -0.75 | PPP2R1B |
| chi-miR-340-5p | XM_018059799.1 | -0.857142857 | PPP2R1B |
| chi-miR-30b-5p | XM_018059799.1 | -0.678571429 | PPP2R1B |
| chi-miR-128-3p | XM_018059799.1 | -0.714285714 | PPP2R1B |
| chi-miR-495-3p | XM_018059799.1 | -0.892857143 | PPP2R1B |
| chi-miR-374b-5p | XM_018059799.1 | -0.821428571 | PPP2R1B |
| chi-miR-499-3p | XM_018059799.1 | -0.928571429 | PPP2R1B |
| chi-miR-30f-5p | XM_018059799.1 | -0.642857143 | PPP2R1B |
| chi-miR-487a-3p | XM_018059799.1 | -0.857142857 | PPP2R1B |
| chi-miR-130b-5p | XM_018059799.1 | -0.821428571 | PPP2R1B |
| chi-miR-496-3p | XM_018059799.1 | -0.928571429 | PPP2R1B |
| chi-miR-181b-3p | XM_018059799.1 | -0.607142857 | PPP2R1B |
| chi-miR-217-3p | XM_018059799.1 | -0.668153105 | PPP2R1B |
| miR-2285-y | XM_018059799.1 | -0.75 | PPP2R1B |
| miR-484-z | XM_018059799.1 | -0.785714286 | PPP2R1B |
| miR-539-y | XM_018059799.1 | -0.928571429 | PPP2R1B |
| miR-9-z | XM_018059799.1 | -0.666693722 | PPP2R1B |
| miR-6119-y | XM_018059799.1 | -0.678571429 | PPP2R1B |
| miR-128-y | XM_018059799.1 | -0.571428571 | PPP2R1B |
| miR-425-x | XM_018059799.1 | -0.785714286 | PPP2R1B |
| miR-493-x | XM_018059799.1 | -0.642857143 | PPP2R1B |
| miR-496-y | XM_018059799.1 | -0.95499371 | PPP2R1B |
| miR-6529-y | XM_018059799.1 | -0.571428571 | PPP2R1B |
| miR-499-y | XM_018059799.1 | -0.668153105 | PPP2R1B |
| miR-2435-x | XM_018059799.1 | -0.792824967 | PPP2R1B |
| miR-485-y | XM_018059799.1 | -0.882918713 | PPP2R1B |
| miR-216-y | XM_018059799.1 | -0.785714286 | PPP2R1B |
| miR-224-y | XM_018059799.1 | -0.866921447 | PPP2R1B |
| miR-664-y | XM_018059799.1 | -0.788110406 | PPP2R1B |
| novel-m0127-5p | XM_018059799.1 | -0.892857143 | PPP2R1B |
| novel-m0021-3p | XM_018059799.1 | -0.756787469 | PPP2R1B |
| novel-m0094-3p | XM_018059799.1 | -0.852436714 | PPP2R1B |
| novel-m0010-3p | XM_018059799.1 | -0.866921447 | PPP2R1B |
| novel-m0053-3p | XM_018059799.1 | -0.579066024 | PPP2R1B |
| miR-1-z | XM_018059799.1 | -0.857142857 | PPP2R1B |
| chi-miR-451-3p | XM_018059799.1 | -0.612372436 | PPP2R1B |
| miR-9-y | XM_018059799.1 | -0.579066024 | PPP2R1B |
| novel-m0131-5p | XM_018059799.1 | -0.535714286 | PPP2R1B |
| chi-miR-145-5p | XM_018060160.1 | -0.571428571 | TGFB2 |
| miR-6215-y | XM_018060160.1 | -0.612372436 | TGFB2 |
| chi-miR-199a-5p | XM_018060160.1 | -0.535714286 | TGFB2 |
| chi-miR-136-5p | XM_018060160.1 | -0.535714286 | TGFB2 |
| chi-miR-200a | XM_018060160.1 | -0.964285714 | TGFB2 |
| chi-miR-200b | XM_018060160.1 | -0.964285714 | TGFB2 |
| chi-miR-200c | XM_018060160.1 | -0.964285714 | TGFB2 |
| chi-miR-141 | XM_018060160.1 | -0.892857143 | TGFB2 |
| chi-miR-301a-3p | XM_018060160.1 | -0.607142857 | TGFB2 |
| chi-miR-429 | XM_018060160.1 | -0.928571429 | TGFB2 |
| chi-miR-29a-3p | XM_018060160.1 | -0.678571429 | TGFB2 |
| chi-miR-30e-3p | XM_018060160.1 | -0.857142857 | TGFB2 |
| chi-miR-33a-5p | XM_018060160.1 | -0.642857143 | TGFB2 |
| chi-miR-7-3p | XM_018060160.1 | -0.892857143 | TGFB2 |
| chi-miR-29b-3p | XM_018060160.1 | -0.678571429 | TGFB2 |
| miR-203-y | XM_018060160.1 | -0.892857143 | TGFB2 |
| miR-2284-x | XM_018060160.1 | -0.714285714 | TGFB2 |
| miR-200-y | XM_018060160.1 | -0.821428571 | TGFB2 |
| miR-3431-x | XM_018060160.1 | -0.75 | TGFB2 |
| miR-142-x | XM_018060160.1 | -0.714285714 | TGFB2 |
| miR-429-y | XM_018060160.1 | -0.928571429 | TGFB2 |
| miR-136-x | XM_018060160.1 | -0.642857143 | TGFB2 |
| miR-2440-y | XM_018060160.1 | -0.821428571 | TGFB2 |
| miR-29-y | XM_018060160.1 | -0.821428571 | TGFB2 |
| miR-205-y | XM_018060160.1 | -0.607142857 | TGFB2 |
| miR-33-x | XM_018060160.1 | -0.642857143 | TGFB2 |
| miR-2284-z | XM_018060160.1 | -0.642857143 | TGFB2 |
| miR-21-y | XM_018060160.1 | -0.714285714 | TGFB2 |
| miR-2285-x | XM_018060160.1 | -0.72074997 | TGFB2 |
| miR-1434-y | XM_018060160.1 | -0.926561646 | TGFB2 |
| miR-4872-x | XM_018060160.1 | -0.534522484 | TGFB2 |
| miR-236-y | XM_018060160.1 | -0.846881215 | TGFB2 |
| miR-6087-x | XM_018060160.1 | -0.801783726 | TGFB2 |
| novel-m0134-5p | XM_018060160.1 | -0.75 | TGFB2 |
| novel-m0028-3p | XM_018060160.1 | -0.704186851 | TGFB2 |
| novel-m0024-5p | XM_018060160.1 | -0.801783726 | TGFB2 |
| chi-miR-130b-3p | XM_018060160.1 | -0.714285714 | TGFB2 |
| chi-miR-2483-3p | XM_018060160.1 | -0.535714286 | TGFB2 |
| novel-m0014-3p | XM_018060160.1 | -0.815374248 | TGFB2 |
| miR-30-y | XM_018060160.1 | -0.714285714 | TGFB2 |
| miR-2448-y | XM_018060160.1 | -0.75 | TGFB2 |
| chi-miR-106b-5p | XM_018061575.1 | -0.555936987 | SMAD1 |
| chi-miR-17-5p | XM_018061575.1 | -0.555936987 | SMAD1 |
| chi-miR-665 | XM_018061575.1 | -0.555936987 | SMAD1 |
| chi-miR-20b | XM_018061575.1 | -0.852436714 | SMAD1 |
| chi-miR-656 | XM_018061575.1 | -0.555936987 | SMAD1 |
| chi-miR-190a-3p | XM_018061575.1 | -0.555936987 | SMAD1 |
| miR-2285-y | XM_018061575.1 | -0.667124385 | SMAD1 |
| miR-665-y | XM_018061575.1 | -0.555936987 | SMAD1 |
| miR-106-x | XM_018061575.1 | -0.815374248 | SMAD1 |
| miR-144-x | XM_018061575.1 | -0.555936987 | SMAD1 |
| novel-m0088-3p | XM_018061575.1 | -0.704186851 | SMAD1 |
| novel-m0021-3p | XM_018061575.1 | -0.579667134 | SMAD1 |
| novel-m0133-3p | XM_018061575.1 | -0.647150229 | SMAD1 |
| chi-miR-421-3p | XM_018061575.1 | -0.852436714 | SMAD1 |
| chi-miR-216b | XM_018061575.1 | -0.704186851 | SMAD1 |

Appendix B

| **MAPK signaling pathway（**26 target genes and 565 target miRNAs**）** | | | |
| --- | --- | --- | --- |
| ID | GeneID | rho | Symbol |
| miR-1949-y | XM_005675512.3 | -0.738768719 | MRAS |
| miR-2115-x | XM_005675512.3 | -0.612372436 | MRAS |
| miR-1290-y | XM_005675512.3 | -0.612372436 | MRAS |
| novel-m0008-5p | XM_005675512.3 | -0.612372436 | MRAS |
| novel-m0025-5p | XM_005675512.3 | -0.612372436 | MRAS |
| miR-328-y | XM_005675512.3 | -0.607142857 | MRAS |
| chi-miR-195-5p | XM_005675512.3 | -0.846881215 | MRAS |
| chi-miR-16a-5p | XM_005675512.3 | -0.678571429 | MRAS |
| chi-miR-200a | XM_005675512.3 | -0.821428571 | MRAS |
| chi-miR-193b-3p | XM_005675512.3 | -0.75 | MRAS |
| chi-miR-22-3p | XM_005675512.3 | -0.785714286 | MRAS |
| chi-miR-146b-3p | XM_005675512.3 | -0.714285714 | MRAS |
| chi-miR-223-3p | XM_005675512.3 | -0.892857143 | MRAS |
| chi-miR-92a-5p | XM_005675512.3 | -0.785714286 | MRAS |
| chi-miR-195-3p | XM_005675512.3 | -0.821428571 | MRAS |
| chi-miR-15a-3p | XM_005675512.3 | -0.778311782 | MRAS |
| miR-24-y | XM_005675512.3 | -0.75 | MRAS |
| miR-16-x | XM_005675512.3 | -0.75 | MRAS |
| miR-193-x | XM_005675512.3 | -0.821428571 | MRAS |
| miR-22-y | XM_005675512.3 | -0.75 | MRAS |
| miR-322-x | XM_005675512.3 | -0.535714286 | MRAS |
| miR-193-y | XM_005675512.3 | -0.821428571 | MRAS |
| miR-195-x | XM_005675512.3 | -0.792824967 | MRAS |
| miR-324-y | XM_005675512.3 | -0.714285714 | MRAS |
| miR-296-x | XM_005675512.3 | -0.535714286 | MRAS |
| miR-6596-x | XM_005675512.3 | -0.892857143 | MRAS |
| miR-361-y | XM_005675512.3 | -0.571428571 | MRAS |
| miR-1306-x | XM_005675512.3 | -0.535714286 | MRAS |
| miR-2285-x | XM_005675512.3 | -0.95499371 | MRAS |
| miR-1434-y | XM_005675512.3 | -0.88949918 | MRAS |
| miR-195-y | XM_005675512.3 | -0.642857143 | MRAS |
| miR-457-x | XM_005675512.3 | -0.714285714 | MRAS |
| miR-223-y | XM_005675512.3 | -0.642857143 | MRAS |
| miR-4306-y | XM_005675512.3 | -0.815374248 | MRAS |
| novel-m0134-5p | XM_005675512.3 | -0.75 | MRAS |
| novel-m0084-5p | XM_005675512.3 | -0.666693722 | MRAS |
| novel-m0137-5p | XM_005675512.3 | -0.846881215 | MRAS |
| novel-m0127-3p | XM_005675512.3 | -0.704186851 | MRAS |
| novel-m0024-5p | XM_005675512.3 | -0.801783726 | MRAS |
| chi-miR-193a | XM_005675512.3 | -0.571428571 | MRAS |
| novel-m0102-3p | XM_005675512.3 | -0.642857143 | MRAS |
| chi-miR-135b-5p | XM_005675512.3 | -0.75 | MRAS |
| chi-miR-1306-5p | XM_005675512.3 | -0.535714286 | MRAS |
| chi-miR-324-3p | XM_005675512.3 | -0.571428571 | MRAS |
| chi-miR-18a-3p | XM_005675512.3 | -0.785714286 | MRAS |
| miR-345-x | XM_005675512.3 | -0.571428571 | MRAS |
| miR-2474-y | XM_005675512.3 | -0.702731221 | MRAS |
| novel-m0100-3p | XM_005675512.3 | -0.857142857 | MRAS |
| chi-miR-410-3p | XM_005677146.3 | -0.607142857 | HSPA6 |
| chi-miR-129-3p | XM_005677146.3 | -0.785714286 | HSPA6 |
| chi-miR-98-3p | XM_005677146.3 | -0.571428571 | HSPA6 |
| chi-miR-30e-5p | XM_005686292.3 | -0.518874522 | IL1R2 |
| chi-miR-532-5p | XM_005686292.3 | -0.592999453 | IL1R2 |
| chi-miR-30c-5p | XM_005686292.3 | -0.630061919 | IL1R2 |
| chi-miR-323a-3p | XM_005686292.3 | -0.741249317 | IL1R2 |
| chi-miR-188-3p | XM_005686292.3 | -0.630061919 | IL1R2 |
| chi-miR-15b-3p | XM_005686292.3 | -0.592999453 | IL1R2 |
| chi-miR-873-5p | XM_005686292.3 | -0.630061919 | IL1R2 |
| miR-532-x | XM_005686292.3 | -0.815374248 | IL1R2 |
| miR-15-y | XM_005686292.3 | -0.704186851 | IL1R2 |
| miR-502-x | XM_005686292.3 | -0.617065013 | IL1R2 |
| miR-664-y | XM_005686292.3 | -0.572502574 | IL1R2 |
| novel-m0061-5p | XM_005686292.3 | -0.592999453 | IL1R2 |
| novel-m0084-3p | XM_005686292.3 | -0.704186851 | IL1R2 |
| chi-miR-217-5p | XM_005691555.3 | -0.642857143 | MAPKAPK5 |
| miR-2312-y | XM_005691555.3 | -0.714285714 | MAPKAPK5 |
| miR-196-y | XM_005691555.3 | -0.535714286 | MAPKAPK5 |
| miR-214-x | XM_005691555.3 | -0.594618725 | MAPKAPK5 |
| miR-1285-y | XM_005693739.1 | -0.678571429 | CACNB1 |
| chi-miR-16b-3p | XM_005695798.3 | -0.892857143 | FLNB |
| miR-495-y | XM_005695798.3 | -0.857142857 | FLNB |
| miR-6529-y | XM_005695798.3 | -0.535714286 | FLNB |
| miR-152-x | XM_005695798.3 | -0.630656224 | FLNB |
| miR-134-x | XM_013966480.2 | -0.571428571 | MAP3K7 |
| miR-1185-y | XM_013966480.2 | -0.678571429 | MAP3K7 |
| chi-miR-542-3p | XM_013966480.2 | -0.535714286 | MAP3K7 |
| miR-544-y | XM_013966480.2 | -0.642857143 | MAP3K7 |
| chi-miR-19b-3p | XM_013967462.2 | -0.678571429 | IL1R1 |
| chi-miR-19a | XM_013967462.2 | -0.785714286 | IL1R1 |
| chi-miR-222-3p | XM_013967462.2 | -0.714285714 | IL1R1 |
| chi-miR-545-3p | XM_013967462.2 | -0.821428571 | IL1R1 |
| chi-miR-491-5p | XM_013967462.2 | -0.607142857 | IL1R1 |
| miR-491-x | XM_013967462.2 | -0.571428571 | IL1R1 |
| miR-1268-x | XM_013967462.2 | -0.778311782 | IL1R1 |
| miR-1273-x | XM_013967462.2 | -0.571428571 | IL1R1 |
| miR-6126-x | XM_013967462.2 | -0.815374248 | IL1R1 |
| novel-m0026-5p | XM_013967462.2 | -0.778311782 | IL1R1 |
| miR-1261-x | XM_013967462.2 | -0.650191085 | IL1R1 |
| miR-1271-y | XM_013967462.2 | -0.571428571 | IL1R1 |
| miR-545-x | XM_013967462.2 | -0.810843716 | IL1R1 |
| novel-m0014-3p | XM_013967462.2 | -0.555936987 | IL1R1 |
| chi-miR-3431-5p | XM_018038577.1 | -0.75 | DAXX |
| miR-3431-x | XM_018038577.1 | -0.642857143 | DAXX |
| miR-497-y | XM_018038577.1 | -0.852436714 | DAXX |
| miR-670-y | XM_018038577.1 | -0.846881215 | DAXX |
| novel-m0118-5p | XM_018040044.1 | -0.579066024 | MAPK8IP3 |
| novel-m0125-5p | XM_018040044.1 | -0.668153105 | MAPK8IP3 |
| chi-miR-125b-5p | XM_018040044.1 | -0.607142857 | MAPK8IP3 |
| chi-miR-195-5p | XM_018040044.1 | -0.846881215 | MAPK8IP3 |
| chi-miR-708-5p | XM_018040044.1 | -0.821428571 | MAPK8IP3 |
| chi-miR-22-3p | XM_018040044.1 | -0.571428571 | MAPK8IP3 |
| chi-miR-361-3p | XM_018040044.1 | -0.75 | MAPK8IP3 |
| chi-miR-92a-5p | XM_018040044.1 | -0.642857143 | MAPK8IP3 |
| miR-27-y | XM_018040044.1 | -0.678571429 | MAPK8IP3 |
| miR-185-x | XM_018040044.1 | -0.642857143 | MAPK8IP3 |
| miR-322-x | XM_018040044.1 | -0.642857143 | MAPK8IP3 |
| miR-195-x | XM_018040044.1 | -0.738768719 | MAPK8IP3 |
| lin-4-x | XM_018040044.1 | -0.607142857 | MAPK8IP3 |
| miR-491-x | XM_018040044.1 | -0.535714286 | MAPK8IP3 |
| miR-6596-x | XM_018040044.1 | -0.821428571 | MAPK8IP3 |
| miR-365-x | XM_018040044.1 | -0.852436714 | MAPK8IP3 |
| miR-4306-y | XM_018040044.1 | -0.630061919 | MAPK8IP3 |
| miR-3656-y | XM_018040044.1 | -0.778311782 | MAPK8IP3 |
| novel-m0127-3p | XM_018040044.1 | -0.555936987 | MAPK8IP3 |
| miR-663-x | XM_018040044.1 | -0.738768719 | MAPK8IP3 |
| chi-miR-874-5p | XM_018040044.1 | -0.678571429 | MAPK8IP3 |
| miR-3432-x | XM_018040044.1 | -0.678571429 | MAPK8IP3 |
| miR-5126-y | XM_018040044.1 | -0.714285714 | MAPK8IP3 |
| novel-m0065-5p | XM_018040044.1 | -0.785714286 | MAPK8IP3 |
| novel-m0125-5p | XM_018040046.1 | -0.534522484 | MAPK8IP3 |
| miR-328-y | XM_018040046.1 | -0.75 | MAPK8IP3 |
| chi-miR-125b-5p | XM_018040046.1 | -0.785714286 | MAPK8IP3 |
| chi-miR-195-5p | XM_018040046.1 | -0.702731221 | MAPK8IP3 |
| chi-miR-16b-5p | XM_018040046.1 | -0.642857143 | MAPK8IP3 |
| chi-miR-708-5p | XM_018040046.1 | -0.714285714 | MAPK8IP3 |
| chi-miR-22-3p | XM_018040046.1 | -0.607142857 | MAPK8IP3 |
| chi-miR-361-3p | XM_018040046.1 | -0.892857143 | MAPK8IP3 |
| chi-miR-491-5p | XM_018040046.1 | -0.642857143 | MAPK8IP3 |
| chi-miR-92a-5p | XM_018040046.1 | -0.571428571 | MAPK8IP3 |
| miR-27-y | XM_018040046.1 | -0.785714286 | MAPK8IP3 |
| miR-185-x | XM_018040046.1 | -0.714285714 | MAPK8IP3 |
| miR-322-x | XM_018040046.1 | -0.821428571 | MAPK8IP3 |
| miR-331-y | XM_018040046.1 | -0.607142857 | MAPK8IP3 |
| miR-195-x | XM_018040046.1 | -0.756787469 | MAPK8IP3 |
| lin-4-x | XM_018040046.1 | -0.785714286 | MAPK8IP3 |
| miR-296-x | XM_018040046.1 | -0.857142857 | MAPK8IP3 |
| miR-491-x | XM_018040046.1 | -0.75 | MAPK8IP3 |
| miR-6596-x | XM_018040046.1 | -0.714285714 | MAPK8IP3 |
| miR-365-x | XM_018040046.1 | -0.88949918 | MAPK8IP3 |
| miR-457-x | XM_018040046.1 | -0.642857143 | MAPK8IP3 |
| miR-4306-y | XM_018040046.1 | -0.704186851 | MAPK8IP3 |
| miR-3656-y | XM_018040046.1 | -0.741249317 | MAPK8IP3 |
| novel-m0127-3p | XM_018040046.1 | -0.741249317 | MAPK8IP3 |
| chi-miR-328-3p | XM_018040046.1 | -0.785714286 | MAPK8IP3 |
| chi-miR-874-5p | XM_018040046.1 | -0.785714286 | MAPK8IP3 |
| miR-744-x | XM_018040046.1 | -0.607142857 | MAPK8IP3 |
| miR-3432-x | XM_018040046.1 | -0.642857143 | MAPK8IP3 |
| miR-5126-y | XM_018040046.1 | -0.785714286 | MAPK8IP3 |
| novel-m0065-5p | XM_018040046.1 | -0.75 | MAPK8IP3 |
| chi-miR-340-5p | XM_018041694.1 | -0.857142857 | DUSP5 |
| chi-miR-128-3p | XM_018041694.1 | -0.571428571 | DUSP5 |
| chi-miR-340-3p | XM_018041694.1 | -0.75 | DUSP5 |
| chi-miR-502b-3p | XM_018041694.1 | -0.821428571 | DUSP5 |
| chi-miR-15b-3p | XM_018041694.1 | -0.714285714 | DUSP5 |
| chi-miR-412-3p | XM_018041694.1 | -0.785714286 | DUSP5 |
| chi-miR-130a-5p | XM_018041694.1 | -0.785714286 | DUSP5 |
| miR-452-x | XM_018041694.1 | -1 | DUSP5 |
| miR-92-y | XM_018041694.1 | -0.607142857 | DUSP5 |
| miR-539-x | XM_018041694.1 | -0.964285714 | DUSP5 |
| miR-210-x | XM_018041694.1 | -0.642857143 | DUSP5 |
| miR-874-y | XM_018041694.1 | -0.785714286 | DUSP5 |
| miR-421-y | XM_018041694.1 | -0.535714286 | DUSP5 |
| miR-665-x | XM_018041694.1 | -0.892857143 | DUSP5 |
| miR-15-y | XM_018041694.1 | -0.821428571 | DUSP5 |
| miR-138-y | XM_018041694.1 | -0.846881215 | DUSP5 |
| novel-m0094-3p | XM_018041694.1 | -0.815374248 | DUSP5 |
| novel-m0075-3p | XM_018041694.1 | -0.866921447 | DUSP5 |
| novel-m0095-3p | XM_018041694.1 | -0.866921447 | DUSP5 |
| chi-miR-421-3p | XM_018041694.1 | -0.571428571 | DUSP5 |
| miR-4286-z | XM_018041770.1 | -0.535714286 | FGFR1 |
| chi-miR-216b | XM_018041770.1 | -0.535714286 | FGFR1 |
| chi-miR-873-5p | XM_018041771.1 | -0.678571429 | FGFR1 |
| miR-183-x | XM_018041771.1 | -0.642857143 | FGFR1 |
| miR-4286-x | XM_018041771.1 | -0.666693722 | FGFR1 |
| miR-4286-y | XM_018041771.1 | -0.738768719 | FGFR1 |
| chi-miR-10b-5p | XM_018046858.1 | -0.857142857 | BRAF |
| chi-miR-30e-5p | XM_018046858.1 | -0.714285714 | BRAF |
| chi-miR-10a-5p | XM_018046858.1 | -0.821428571 | BRAF |
| chi-miR-369-3p | XM_018046858.1 | -0.642857143 | BRAF |
| chi-miR-335-5p | XM_018046858.1 | -0.642857143 | BRAF |
| chi-miR-494 | XM_018046858.1 | -0.714285714 | BRAF |
| chi-miR-96 | XM_018046858.1 | -0.785714286 | BRAF |
| chi-miR-655 | XM_018046858.1 | -0.607142857 | BRAF |
| chi-miR-1271-5p | XM_018046858.1 | -0.535714286 | BRAF |
| chi-miR-411a-3p | XM_018046858.1 | -0.535714286 | BRAF |
| chi-miR-660 | XM_018046858.1 | -0.642857143 | BRAF |
| chi-miR-340-5p | XM_018046858.1 | -0.678571429 | BRAF |
| chi-miR-499-5p | XM_018046858.1 | -0.642857143 | BRAF |
| chi-miR-335-3p | XM_018046858.1 | -0.714285714 | BRAF |
| chi-miR-128-3p | XM_018046858.1 | -0.535714286 | BRAF |
| chi-miR-3959-3p | XM_018046858.1 | -0.678571429 | BRAF |
| chi-miR-374b-5p | XM_018046858.1 | -0.785714286 | BRAF |
| chi-miR-379-3p | XM_018046858.1 | -0.642857143 | BRAF |
| chi-miR-382-3p | XM_018046858.1 | -0.642857143 | BRAF |
| chi-miR-376e-3p | XM_018046858.1 | -0.642857143 | BRAF |
| chi-miR-374a-3p | XM_018046858.1 | -0.571428571 | BRAF |
| chi-miR-376a | XM_018046858.1 | -0.535714286 | BRAF |
| chi-miR-329b-3p | XM_018046858.1 | -0.642857143 | BRAF |
| chi-miR-323a-3p | XM_018046858.1 | -0.642857143 | BRAF |
| chi-miR-154b-5p | XM_018046858.1 | -0.607142857 | BRAF |
| chi-miR-208b | XM_018046858.1 | -0.630061919 | BRAF |
| chi-miR-483 | XM_018046858.1 | -0.607142857 | BRAF |
| chi-miR-505-3p | XM_018046858.1 | -0.714285714 | BRAF |
| chi-miR-377 | XM_018046858.1 | -0.714285714 | BRAF |
| chi-miR-10a-3p | XM_018046858.1 | -0.642857143 | BRAF |
| chi-miR-656 | XM_018046858.1 | -0.678571429 | BRAF |
| chi-miR-345-3p | XM_018046858.1 | -0.642857143 | BRAF |
| chi-miR-134 | XM_018046858.1 | -0.607142857 | BRAF |
| chi-miR-129-5p | XM_018046858.1 | -0.714285714 | BRAF |
| chi-miR-224-3p | XM_018046858.1 | -0.857142857 | BRAF |
| chi-miR-329a-3p | XM_018046858.1 | -0.642857143 | BRAF |
| chi-miR-3955-3p | XM_018046858.1 | -0.607142857 | BRAF |
| chi-miR-18b-5p | XM_018046858.1 | -0.535714286 | BRAF |
| chi-miR-16b-3p | XM_018046858.1 | -0.714285714 | BRAF |
| chi-miR-487b-5p | XM_018046858.1 | -0.666693722 | BRAF |
| chi-miR-873-5p | XM_018046858.1 | -0.678571429 | BRAF |
| chi-miR-181b-3p | XM_018046858.1 | -0.642857143 | BRAF |
| chi-miR-34b-3p | XM_018046858.1 | -0.810843716 | BRAF |
| chi-miR-130a-5p | XM_018046858.1 | -0.892857143 | BRAF |
| chi-miR-34c-3p | XM_018046858.1 | -0.785714286 | BRAF |
| chi-miR-450-3p | XM_018046858.1 | -0.535714286 | BRAF |
| miR-10-x | XM_018046858.1 | -0.821428571 | BRAF |
| miR-2285-y | XM_018046858.1 | -0.714285714 | BRAF |
| miR-411-y | XM_018046858.1 | -0.642857143 | BRAF |
| miR-183-x | XM_018046858.1 | -0.785714286 | BRAF |
| miR-335-x | XM_018046858.1 | -0.678571429 | BRAF |
| miR-2387-x | XM_018046858.1 | -0.607142857 | BRAF |
| miR-6119-y | XM_018046858.1 | -0.571428571 | BRAF |
| miR-3959-y | XM_018046858.1 | -0.714285714 | BRAF |
| miR-379-y | XM_018046858.1 | -0.535714286 | BRAF |
| miR-376-x | XM_018046858.1 | -0.642857143 | BRAF |
| miR-329-y | XM_018046858.1 | -0.642857143 | BRAF |
| miR-154-x | XM_018046858.1 | -0.714285714 | BRAF |
| miR-335-y | XM_018046858.1 | -0.666693722 | BRAF |
| miR-539-x | XM_018046858.1 | -0.714285714 | BRAF |
| miR-323-y | XM_018046858.1 | -0.678571429 | BRAF |
| miR-154-y | XM_018046858.1 | -0.607142857 | BRAF |
| miR-874-y | XM_018046858.1 | -0.642857143 | BRAF |
| miR-7862-y | XM_018046858.1 | -0.785714286 | BRAF |
| miR-382-y | XM_018046858.1 | -0.607142857 | BRAF |
| miR-483-y | XM_018046858.1 | -0.558581227 | BRAF |
| miR-129-x | XM_018046858.1 | -0.810843716 | BRAF |
| miR-664-y | XM_018046858.1 | -0.669893845 | BRAF |
| miR-1264-y | XM_018046858.1 | -0.579066024 | BRAF |
| novel-m0088-3p | XM_018046858.1 | -0.678571429 | BRAF |
| novel-m0061-5p | XM_018046858.1 | -0.607142857 | BRAF |
| novel-m0052-5p | XM_018046858.1 | -0.642857143 | BRAF |
| novel-m0079-3p | XM_018046858.1 | -0.630488325 | BRAF |
| novel-m0010-3p | XM_018046858.1 | -0.630488325 | BRAF |
| miR-133-x | XM_018046858.1 | -0.571428571 | BRAF |
| miR-4755-y | XM_018046858.1 | -0.612372436 | BRAF |
| miR-4755-y | XM_018046858.1 | -0.612372436 | BRAF |
| let-7-z | XM_018046858.1 | -0.729002126 | BRAF |
| miR-3955-y | XM_018046858.1 | -0.610785565 | BRAF |
| chi-miR-3431-3p | XM_018046858.1 | -0.857142857 | BRAF |
| miR-224-x | XM_018046858.1 | -0.607142857 | BRAF |
| miR-105-x | XM_018046858.1 | -0.571428571 | BRAF |
| miR-508-x | XM_018046858.1 | -0.592999453 | BRAF |
| chi-miR-182 | XM_018046858.1 | -0.642857143 | BRAF |
| miR-448-y | XM_018046858.1 | -0.778311782 | BRAF |
| miR-186-x | XM_018048466.1 | -0.534522484 | DUSP16 |
| let-7-z | XM_018048466.1 | -0.516046847 | DUSP16 |
| miR-205-y | XM_018048466.1 | -0.579066024 | DUSP16 |
| miR-143-y | XM_018049972.1 | -0.821428571 | MEF2C |
| miR-126-x | XM_018049972.1 | -0.642857143 | MEF2C |
| miR-365-y | XM_018049972.1 | -0.642857143 | MEF2C |
| miR-3607-y | XM_018049972.1 | -0.612372436 | MEF2C |
| novel-m0077-3p | XM_018049972.1 | -0.612372436 | MEF2C |
| chi-miR-200a | XM_018049972.1 | -1 | MEF2C |
| chi-miR-320-3p | XM_018049972.1 | -0.857142857 | MEF2C |
| chi-miR-141 | XM_018049972.1 | -0.964285714 | MEF2C |
| chi-miR-190a-5p | XM_018049972.1 | -0.607142857 | MEF2C |
| chi-miR-28-3p | XM_018049972.1 | -0.857142857 | MEF2C |
| chi-miR-30e-3p | XM_018049972.1 | -0.785714286 | MEF2C |
| chi-miR-33a-5p | XM_018049972.1 | -0.571428571 | MEF2C |
| chi-miR-545-5p | XM_018049972.1 | -0.535714286 | MEF2C |
| chi-miR-365-3p | XM_018049972.1 | -0.642857143 | MEF2C |
| chi-miR-186-3p | XM_018049972.1 | -0.714285714 | MEF2C |
| chi-miR-223-3p | XM_018049972.1 | -0.857142857 | MEF2C |
| chi-miR-33b-5p | XM_018049972.1 | -0.821428571 | MEF2C |
| chi-miR-1468-3p | XM_018049972.1 | -0.774806218 | MEF2C |
| miR-203-y | XM_018049972.1 | -0.964285714 | MEF2C |
| miR-151-y | XM_018049972.1 | -0.928571429 | MEF2C |
| miR-141-y | XM_018049972.1 | -1 | MEF2C |
| miR-2484-y | XM_018049972.1 | -0.821428571 | MEF2C |
| miR-1277-x | XM_018049972.1 | -0.535714286 | MEF2C |
| miR-1384-y | XM_018049972.1 | -0.964285714 | MEF2C |
| miR-28-y | XM_018049972.1 | -0.75 | MEF2C |
| miR-2440-y | XM_018049972.1 | -0.857142857 | MEF2C |
| miR-205-y | XM_018049972.1 | -0.571428571 | MEF2C |
| miR-497-y | XM_018049972.1 | -0.963624112 | MEF2C |
| miR-33-x | XM_018049972.1 | -0.607142857 | MEF2C |
| miR-330-x | XM_018049972.1 | -0.857142857 | MEF2C |
| miR-1434-y | XM_018049972.1 | -0.963624112 | MEF2C |
| miR-2311-x | XM_018049972.1 | -0.828862466 | MEF2C |
| miR-2331-y | XM_018049972.1 | -0.785714286 | MEF2C |
| miR-4429-x | XM_018049972.1 | -0.857142857 | MEF2C |
| miR-223-y | XM_018049972.1 | -0.642857143 | MEF2C |
| miR-4872-x | XM_018049972.1 | -0.668153105 | MEF2C |
| miR-8191-x | XM_018049972.1 | -0.906326967 | MEF2C |
| miR-6536-x | XM_018049972.1 | -0.756787469 | MEF2C |
| novel-m0106-5p | XM_018049972.1 | -0.630061919 | MEF2C |
| miR-1271-y | XM_018049972.1 | -0.535714286 | MEF2C |
| miR-670-y | XM_018049972.1 | -0.846881215 | MEF2C |
| miR-545-x | XM_018049972.1 | -0.630656224 | MEF2C |
| novel-m0014-3p | XM_018049972.1 | -0.741249317 | MEF2C |
| chi-miR-1248-5p | XM_018049972.1 | -0.518874522 | MEF2C |
| miR-30-y | XM_018049972.1 | -0.607142857 | MEF2C |
| chi-miR-494 | XM_018052436.1 | -0.821428571 | NTRK2 |
| chi-miR-340-5p | XM_018052436.1 | -0.642857143 | NTRK2 |
| chi-miR-499-5p | XM_018052436.1 | -0.857142857 | NTRK2 |
| chi-miR-432-5p | XM_018052436.1 | -0.535714286 | NTRK2 |
| chi-miR-544-5p | XM_018052436.1 | -0.821428571 | NTRK2 |
| chi-miR-499-3p | XM_018052436.1 | -0.857142857 | NTRK2 |
| chi-miR-329b-3p | XM_018052436.1 | -0.75 | NTRK2 |
| chi-miR-1197-3p | XM_018052436.1 | -0.642857143 | NTRK2 |
| chi-miR-301a-5p | XM_018052436.1 | -0.642857143 | NTRK2 |
| chi-miR-329a-3p | XM_018052436.1 | -0.75 | NTRK2 |
| chi-miR-1271-3p | XM_018052436.1 | -0.610785565 | NTRK2 |
| chi-miR-181b-3p | XM_018052436.1 | -0.571428571 | NTRK2 |
| chi-miR-450-3p | XM_018052436.1 | -0.535714286 | NTRK2 |
| miR-486-x | XM_018052436.1 | -0.785714286 | NTRK2 |
| miR-299-y | XM_018052436.1 | -0.714285714 | NTRK2 |
| miR-214-y | XM_018052436.1 | -0.571428571 | NTRK2 |
| miR-2285-y | XM_018052436.1 | -0.642857143 | NTRK2 |
| miR-92-y | XM_018052436.1 | -0.607142857 | NTRK2 |
| miR-183-x | XM_018052436.1 | -0.714285714 | NTRK2 |
| miR-7977-x | XM_018052436.1 | -0.678571429 | NTRK2 |
| miR-6119-y | XM_018052436.1 | -0.678571429 | NTRK2 |
| miR-2284-y | XM_018052436.1 | -0.535714286 | NTRK2 |
| miR-329-y | XM_018052436.1 | -0.75 | NTRK2 |
| miR-499-x | XM_018052436.1 | -0.821428571 | NTRK2 |
| miR-340-x | XM_018052436.1 | -0.75 | NTRK2 |
| miR-499-y | XM_018052436.1 | -0.534522484 | NTRK2 |
| miR-362-y | XM_018052436.1 | -0.678571429 | NTRK2 |
| miR-544-x | XM_018052436.1 | -0.810843716 | NTRK2 |
| miR-490-y | XM_018052436.1 | -0.610785565 | NTRK2 |
| miR-664-y | XM_018052436.1 | -0.650191085 | NTRK2 |
| novel-m0127-5p | XM_018052436.1 | -0.678571429 | NTRK2 |
| novel-m0112-5p | XM_018052436.1 | -0.642857143 | NTRK2 |
| novel-m0089-5p | XM_018052436.1 | -0.642857143 | NTRK2 |
| novel-m0007-5p | XM_018052436.1 | -0.610785565 | NTRK2 |
| novel-m0048-3p | XM_018052436.1 | -0.807813166 | NTRK2 |
| novel-m0075-3p | XM_018052436.1 | -0.650191085 | NTRK2 |
| novel-m0095-3p | XM_018052436.1 | -0.650191085 | NTRK2 |
| novel-m0097-3p | XM_018052436.1 | -0.534522484 | NTRK2 |
| chi-miR-145-3p | XM_018052436.1 | -0.607142857 | NTRK2 |
| miR-186-x | XM_018052436.1 | -0.714285714 | NTRK2 |
| chi-miR-3431-3p | XM_018052436.1 | -0.642857143 | NTRK2 |
| chi-miR-421-3p | XM_018052436.1 | -0.571428571 | NTRK2 |
| chi-miR-411a-5p | XM_018054403.1 | -0.792824967 | PLA2G4E |
| chi-miR-148a-3p | XM_018054403.1 | -0.558581227 | PLA2G4E |
| chi-miR-30e-5p | XM_018054403.1 | -0.504524979 | PLA2G4E |
| chi-miR-369-3p | XM_018054403.1 | -0.702731221 | PLA2G4E |
| chi-miR-335-5p | XM_018054403.1 | -0.702731221 | PLA2G4E |
| chi-miR-30c-5p | XM_018054403.1 | -0.612637475 | PLA2G4E |
| chi-miR-1271-5p | XM_018054403.1 | -0.522543728 | PLA2G4E |
| chi-miR-340-5p | XM_018054403.1 | -0.666693722 | PLA2G4E |
| chi-miR-335-3p | XM_018054403.1 | -0.630656224 | PLA2G4E |
| chi-miR-214-3p | XM_018054403.1 | -0.630656224 | PLA2G4E |
| chi-miR-34c-5p | XM_018054403.1 | -0.666693722 | PLA2G4E |
| chi-miR-487a-3p | XM_018054403.1 | -0.666693722 | PLA2G4E |
| chi-miR-362-3p | XM_018054403.1 | -0.540562478 | PLA2G4E |
| chi-miR-154a-3p | XM_018054403.1 | -0.702731221 | PLA2G4E |
| chi-miR-323a-3p | XM_018054403.1 | -0.666693722 | PLA2G4E |
| chi-miR-208b | XM_018054403.1 | -0.654462893 | PLA2G4E |
| chi-miR-323b | XM_018054403.1 | -0.666693722 | PLA2G4E |
| chi-miR-496-3p | XM_018054403.1 | -0.702731221 | PLA2G4E |
| chi-miR-204-5p | XM_018054403.1 | -0.666693722 | PLA2G4E |
| chi-miR-345-3p | XM_018054403.1 | -0.684712472 | PLA2G4E |
| chi-miR-153 | XM_018054403.1 | -0.522543728 | PLA2G4E |
| chi-miR-301a-5p | XM_018054403.1 | -0.666693722 | PLA2G4E |
| chi-miR-15b-3p | XM_018054403.1 | -0.522543728 | PLA2G4E |
| chi-miR-181b-3p | XM_018054403.1 | -0.684712472 | PLA2G4E |
| chi-miR-450-3p | XM_018054403.1 | -0.558581227 | PLA2G4E |
| miR-299-y | XM_018054403.1 | -0.702731221 | PLA2G4E |
| miR-450-y | XM_018054403.1 | -0.864899964 | PLA2G4E |
| miR-502-y | XM_018054403.1 | -0.792824967 | PLA2G4E |
| miR-500-y | XM_018054403.1 | -0.792824967 | PLA2G4E |
| miR-335-x | XM_018054403.1 | -0.666693722 | PLA2G4E |
| miR-539-y | XM_018054403.1 | -0.702731221 | PLA2G4E |
| miR-369-y | XM_018054403.1 | -0.792824967 | PLA2G4E |
| miR-134-x | XM_018054403.1 | -0.882918713 | PLA2G4E |
| miR-335-y | XM_018054403.1 | -0.690909091 | PLA2G4E |
| miR-2355-x | XM_018054403.1 | -0.504524979 | PLA2G4E |
| miR-345-y | XM_018054403.1 | -0.684712472 | PLA2G4E |
| miR-154-y | XM_018054403.1 | -0.666693722 | PLA2G4E |
| miR-7862-y | XM_018054403.1 | -0.612637475 | PLA2G4E |
| miR-204-x | XM_018054403.1 | -0.774806218 | PLA2G4E |
| miR-496-y | XM_018054403.1 | -0.690909091 | PLA2G4E |
| miR-506-x | XM_018054403.1 | -0.810843716 | PLA2G4E |
| miR-362-y | XM_018054403.1 | -0.504524979 | PLA2G4E |
| miR-15-y | XM_018054403.1 | -0.630656224 | PLA2G4E |
| miR-544-x | XM_018054403.1 | -0.836363636 | PLA2G4E |
| novel-m0033-5p | XM_018054403.1 | -0.666693722 | PLA2G4E |
| novel-m0112-5p | XM_018054403.1 | -0.666693722 | PLA2G4E |
| novel-m0048-3p | XM_018054403.1 | -0.526848337 | PLA2G4E |
| novel-m0079-3p | XM_018054403.1 | -0.586491545 | PLA2G4E |
| chi-miR-543-5p | XM_018054403.1 | -0.691860773 | PLA2G4E |
| chi-miR-224-5p | XM_018054403.1 | -0.522543728 | PLA2G4E |
| miR-224-x | XM_018054403.1 | -0.738768719 | PLA2G4E |
| miR-377-y | XM_018054403.1 | -0.635763953 | PLA2G4E |
| chi-miR-10b-5p | XM_018054406.1 | -0.892857143 | LOC102175216 |
| chi-miR-148a-3p | XM_018054406.1 | -0.75 | LOC102175216 |
| chi-miR-10a-5p | XM_018054406.1 | -0.928571429 | LOC102175216 |
| chi-miR-148b-3p | XM_018054406.1 | -0.642857143 | LOC102175216 |
| chi-miR-214-3p | XM_018054406.1 | -0.964285714 | LOC102175216 |
| chi-miR-34c-5p | XM_018054406.1 | -0.964285714 | LOC102175216 |
| chi-miR-329b-3p | XM_018054406.1 | -0.964285714 | LOC102175216 |
| chi-miR-362-3p | XM_018054406.1 | -0.964285714 | LOC102175216 |
| chi-miR-329a-3p | XM_018054406.1 | -0.964285714 | LOC102175216 |
| miR-10-x | XM_018054406.1 | -0.928571429 | LOC102175216 |
| miR-214-y | XM_018054406.1 | -0.857142857 | LOC102175216 |
| miR-329-y | XM_018054406.1 | -0.964285714 | LOC102175216 |
| miR-362-y | XM_018054406.1 | -0.857142857 | LOC102175216 |
| novel-m0097-3p | XM_018054406.1 | -0.757240185 | LOC102175216 |
| miR-133-x | XM_018054406.1 | -0.678571429 | LOC102175216 |
| chi-miR-10b-5p | XM_018054409.1 | -0.928571429 | LOC102175216 |
| chi-miR-148a-3p | XM_018054409.1 | -0.821428571 | LOC102175216 |
| chi-miR-10a-5p | XM_018054409.1 | -0.892857143 | LOC102175216 |
| chi-miR-148b-3p | XM_018054409.1 | -0.535714286 | LOC102175216 |
| chi-miR-214-3p | XM_018054409.1 | -0.857142857 | LOC102175216 |
| chi-miR-34c-5p | XM_018054409.1 | -0.714285714 | LOC102175216 |
| chi-miR-329b-3p | XM_018054409.1 | -0.857142857 | LOC102175216 |
| chi-miR-362-3p | XM_018054409.1 | -0.785714286 | LOC102175216 |
| chi-miR-329a-3p | XM_018054409.1 | -0.857142857 | LOC102175216 |
| miR-10-x | XM_018054409.1 | -0.892857143 | LOC102175216 |
| miR-214-y | XM_018054409.1 | -0.678571429 | LOC102175216 |
| miR-329-y | XM_018054409.1 | -0.857142857 | LOC102175216 |
| miR-362-y | XM_018054409.1 | -0.678571429 | LOC102175216 |
| miR-133-x | XM_018054409.1 | -0.857142857 | LOC102175216 |
| chi-miR-21-5p | XM_018058285.1 | -0.810843716 | STK3 |
| chi-miR-30e-3p | XM_018058285.1 | -0.900937463 | STK3 |
| chi-miR-545-3p | XM_018058285.1 | -0.882918713 | STK3 |
| chi-miR-7-3p | XM_018058285.1 | -0.95499371 | STK3 |
| chi-miR-27b-5p | XM_018058285.1 | -0.846881215 | STK3 |
| chi-miR-671-3p | XM_018058285.1 | -0.738768719 | STK3 |
| miR-142-x | XM_018058285.1 | -0.846881215 | STK3 |
| miR-1386-x | XM_018058285.1 | -0.95499371 | STK3 |
| miR-1277-x | XM_018058285.1 | -0.666693722 | STK3 |
| miR-186-y | XM_018058285.1 | -0.727272727 | STK3 |
| miR-671-y | XM_018058285.1 | -0.729258652 | STK3 |
| miR-3976-x | XM_018058285.1 | -0.786566506 | STK3 |
| novel-m0121-3p | XM_018058285.1 | -0.781818182 | STK3 |
| miR-545-x | XM_018058285.1 | -0.836363636 | STK3 |
| chi-miR-29b-5p | XM_018058285.1 | -0.872727273 | STK3 |
| miR-30-y | XM_018058285.1 | -0.882918713 | STK3 |
| chi-miR-145-5p | XM_018060160.1 | -0.571428571 | TGFB2 |
| miR-6215-y | XM_018060160.1 | -0.612372436 | TGFB2 |
| chi-miR-199a-5p | XM_018060160.1 | -0.535714286 | TGFB2 |
| chi-miR-136-5p | XM_018060160.1 | -0.535714286 | TGFB2 |
| chi-miR-200a | XM_018060160.1 | -0.964285714 | TGFB2 |
| chi-miR-200b | XM_018060160.1 | -0.964285714 | TGFB2 |
| chi-miR-200c | XM_018060160.1 | -0.964285714 | TGFB2 |
| chi-miR-141 | XM_018060160.1 | -0.892857143 | TGFB2 |
| chi-miR-301a-3p | XM_018060160.1 | -0.607142857 | TGFB2 |
| chi-miR-429 | XM_018060160.1 | -0.928571429 | TGFB2 |
| chi-miR-29a-3p | XM_018060160.1 | -0.678571429 | TGFB2 |
| chi-miR-30e-3p | XM_018060160.1 | -0.857142857 | TGFB2 |
| chi-miR-33a-5p | XM_018060160.1 | -0.642857143 | TGFB2 |
| chi-miR-7-3p | XM_018060160.1 | -0.892857143 | TGFB2 |
| chi-miR-29b-3p | XM_018060160.1 | -0.678571429 | TGFB2 |
| miR-203-y | XM_018060160.1 | -0.892857143 | TGFB2 |
| miR-2284-x | XM_018060160.1 | -0.714285714 | TGFB2 |
| miR-200-y | XM_018060160.1 | -0.821428571 | TGFB2 |
| miR-3431-x | XM_018060160.1 | -0.75 | TGFB2 |
| miR-142-x | XM_018060160.1 | -0.714285714 | TGFB2 |
| miR-429-y | XM_018060160.1 | -0.928571429 | TGFB2 |
| miR-136-x | XM_018060160.1 | -0.642857143 | TGFB2 |
| miR-2440-y | XM_018060160.1 | -0.821428571 | TGFB2 |
| miR-29-y | XM_018060160.1 | -0.821428571 | TGFB2 |
| miR-205-y | XM_018060160.1 | -0.607142857 | TGFB2 |
| miR-33-x | XM_018060160.1 | -0.642857143 | TGFB2 |
| miR-2284-z | XM_018060160.1 | -0.642857143 | TGFB2 |
| miR-21-y | XM_018060160.1 | -0.714285714 | TGFB2 |
| miR-2285-x | XM_018060160.1 | -0.72074997 | TGFB2 |
| miR-1434-y | XM_018060160.1 | -0.926561646 | TGFB2 |
| miR-4872-x | XM_018060160.1 | -0.534522484 | TGFB2 |
| miR-236-y | XM_018060160.1 | -0.846881215 | TGFB2 |
| miR-6087-x | XM_018060160.1 | -0.801783726 | TGFB2 |
| novel-m0134-5p | XM_018060160.1 | -0.75 | TGFB2 |
| novel-m0028-3p | XM_018060160.1 | -0.704186851 | TGFB2 |
| novel-m0024-5p | XM_018060160.1 | -0.801783726 | TGFB2 |
| chi-miR-130b-3p | XM_018060160.1 | -0.714285714 | TGFB2 |
| chi-miR-2483-3p | XM_018060160.1 | -0.535714286 | TGFB2 |
| novel-m0014-3p | XM_018060160.1 | -0.815374248 | TGFB2 |
| miR-30-y | XM_018060160.1 | -0.714285714 | TGFB2 |
| miR-2448-y | XM_018060160.1 | -0.75 | TGFB2 |
| chi-miR-214-3p | XM_018062408.1 | -0.667124385 | AKT2 |
| chi-miR-544-5p | XM_018062408.1 | -0.630061919 | AKT2 |
| chi-miR-28-5p | XM_018062408.1 | -0.592999453 | AKT2 |
| chi-miR-374a-3p | XM_018062408.1 | -0.704186851 | AKT2 |
| chi-miR-34c-5p | XM_018062408.1 | -0.704186851 | AKT2 |
| chi-miR-362-3p | XM_018062408.1 | -0.518874522 | AKT2 |
| chi-miR-502b-3p | XM_018062408.1 | -0.667124385 | AKT2 |
| chi-miR-876-3p | XM_018062408.1 | -0.572502574 | AKT2 |
| miR-2285-y | XM_018062408.1 | -0.852436714 | AKT2 |
| miR-450-y | XM_018062408.1 | -0.852436714 | AKT2 |
| miR-615-y | XM_018062408.1 | -0.778311782 | AKT2 |
| miR-485-x | XM_018062408.1 | -0.630061919 | AKT2 |
| miR-7859-y | XM_018062408.1 | -0.518874522 | AKT2 |
| miR-541-y | XM_018062408.1 | -0.630061919 | AKT2 |
| miR-219-y | XM_018062408.1 | -0.555936987 | AKT2 |
| miR-362-y | XM_018062408.1 | -0.592999453 | AKT2 |
| novel-m0127-5p | XM_018062408.1 | -0.667124385 | AKT2 |
| miR-3548-y | XM_018062408.1 | -0.730769231 | AKT2 |
| chi-miR-148b-3p | XM_018063974.1 | -0.607142857 | CACNG5 |
| miR-4286-z | XM_018063974.1 | -0.607142857 | CACNG5 |
| novel-m0037-3p | XM_018063974.1 | -0.534522484 | CACNG5 |
| novel-m0053-3p | XM_018063974.1 | -0.534522484 | CACNG5 |
| chi-miR-193a | XM_018063974.1 | -0.571428571 | CACNG5 |
| miR-802-x | XM_018063974.1 | -0.610785565 | CACNG5 |
| miR-1599-y | XM_018063974.1 | -0.882918713 | CACNG5 |
| miR-124-x | XM_018063974.1 | -0.612372436 | CACNG5 |
| chi-miR-330-5p | XM_018063974.1 | -0.75 | CACNG5 |
| miR-32-x | XM_018063974.1 | -0.607142857 | CACNG5 |
| miR-628-x | XM_018063974.1 | -0.522543728 | CACNG5 |
| chi-miR-628-5p | XM_018063974.1 | -0.75 | CACNG5 |
| chi-miR-2318 | XM_018063974.1 | -0.964285714 | CACNG5 |
| chi-miR-150 | XM_018063976.1 | -0.607142857 | CACNG1 |
| novel-m0024-5p | XM_018063976.1 | -0.757240185 | CACNG1 |
| chi-miR-20a-5p | XM_018063976.1 | -0.785714286 | CACNG1 |
| miR-1247-x | XM_018063976.1 | -0.576599976 | CACNG1 |
| miR-1343-y | XM_018064993.1 | -0.512271764 | CACNB1 |
| miR-1343-y | XM_018064993.1 | -0.512271764 | CACNB1 |
| miR-1343-y | XM_018064993.1 | -0.512271764 | CACNB1 |
| chi-miR-340-5p | XM_018065290.1 | -0.571428571 | MAPT |
| chi-miR-197-3p | XM_018065290.1 | -0.642857143 | MAPT |
| chi-miR-1197-3p | XM_018065290.1 | -0.571428571 | MAPT |
| chi-miR-504 | XM_018065290.1 | -0.678571429 | MAPT |
| chi-miR-20b | XM_018065290.1 | -0.571428571 | MAPT |
| chi-miR-1307-5p | XM_018065290.1 | -0.607142857 | MAPT |
| chi-miR-181b-3p | XM_018065290.1 | -0.892857143 | MAPT |
| miR-503-x | XM_018065290.1 | -0.571428571 | MAPT |
| miR-214-y | XM_018065290.1 | -0.714285714 | MAPT |
| miR-615-y | XM_018065290.1 | -0.821428571 | MAPT |
| miR-485-x | XM_018065290.1 | -0.678571429 | MAPT |
| miR-132-y | XM_018065290.1 | -0.535714286 | MAPT |
| miR-197-y | XM_018065290.1 | -0.607142857 | MAPT |
| miR-219-x | XM_018065290.1 | -0.571428571 | MAPT |
| novel-m0112-5p | XM_018065290.1 | -0.571428571 | MAPT |
| miR-105-x | XM_018065290.1 | -0.607142857 | MAPT |
| chi-miR-219 | XM_018065290.1 | -0.666693722 | MAPT |
| miR-17-y | XM_018065290.1 | -0.571428571 | MAPT |
| chi-miR-24-3p | XM_018065330.1 | -0.821428571 | MAP3K3 |
| novel-m0040-5p | XM_018065330.1 | -0.612372436 | MAP3K3 |
| novel-m0024-5p | XM_018065330.1 | -0.801783726 | MAP3K3 |
| chi-miR-193a | XM_018065330.1 | -0.857142857 | MAP3K3 |
| miR-194-x | XM_018065330.1 | -0.535714286 | MAP3K3 |
| miR-663-x | XM_018065330.1 | -0.630656224 | MAP3K3 |
| chi-miR-330-5p | XM_018065330.1 | -0.642857143 | MAP3K3 |
| miR-22-x | XM_018065330.1 | -0.678571429 | MAP3K3 |
| miR-7857-y | XM_018065330.1 | -0.821428571 | MAP3K3 |
| chi-miR-133a-3p | XM_018065720.1 | -0.928571429 | DUSP1 |
| chi-miR-495-3p | XM_018065720.1 | -0.964285714 | DUSP1 |
| chi-miR-133b | XM_018065720.1 | -0.821428571 | DUSP1 |
| chi-miR-505-3p | XM_018065720.1 | -0.75 | DUSP1 |
| chi-miR-656 | XM_018065720.1 | -0.964285714 | DUSP1 |
| miR-450-y | XM_018065720.1 | -0.75 | DUSP1 |
| miR-3959-y | XM_018065720.1 | -0.785714286 | DUSP1 |
| novel-m0079-3p | XM_018065720.1 | -0.788110406 | DUSP1 |
| miR-133-y | XM_018065720.1 | -0.928571429 | DUSP1 |
| miR-133-z | XM_018065720.1 | -0.785714286 | DUSP1 |
| miR-411-x | XM_018065720.1 | -0.678571429 | DUSP1 |
| novel-m0133-3p | XM_018065720.1 | -0.801783726 | DUSP1 |
